# Collective homeostasis of condensation-prone proteins via their mRNAs

**DOI:** 10.1038/s41586-025-09568-w

**Published:** 2025-09-24

**Authors:** Rupert Faraway, Neve Costello Heaven, Holly Digby, Klara Kuret Hodnik, Jure Rebselj, Oscar G. Wilkins, Anob M. Chakrabarti, Ira A. Iosub, Neža Vadnjal, Rhys Dore, Lea Knez, Stefan L. Ameres, Clemens Plaschka, Jernej Ule

**Affiliations:** 1 The Francis Crick Institute, London, UK; 2 UK Dementia Research Institute at King’s College London, London, UK; 3 Department of Basic and Clinical Neuroscience, Institute of Psychiatry Psychology and Neuroscience, King’s College London, London, UK; 4 Max Perutz Labs, University of Vienna, Vienna BioCenter, Vienna, Austria; 5 Research Institute of Molecular Pathology, Vienna BioCenter, Vienna, Austria; 6 National Institute of Chemistry, Ljubljana, Slovenia; 7 Department of Neuromuscular Diseases, UCL Queen Square Institute of Neurology, UCL, London, UK; 8 UCL Respiratory, Division of Medicine, University College London, London, UK

**Keywords:** RNA, Nuclear speckles, RNA transport

## Abstract

The concentration of proteins containing intrinsically disordered regions must be tightly controlled to maintain cellular homeostasis^1,2^. However, mechanisms for collective control of these proteins, which tend to localize to membraneless condensates, are less understood than pathways mediated by membrane-bound organelles^3,4^. Here we report ‘interstasis’, a homeostatic mechanism in which increased concentration of proteins within RNA–protein condensates induces the sequestration of their own mRNAs. The selectivity of interstatic mRNA capture relies on the structure of the genetic code and conserved codon biases, which ensure that similar multivalent RNA regions encode similar low-complexity domains. For example, arginine-enriched mixed charge domains (R-MCDs) tend to be encoded by repetitive purine-rich sequences in mRNAs. Accumulation of proteins containing R-MCDs increases the cohesion of nuclear speckles, which induces selective capture of purine-rich multivalent mRNAs. The multivalent regions are bound by specific RNA-binding proteins, including TRA2 proteins, which relocalize to speckles upon interstasis to promote selective mRNA capture. CLK-mediated phosphorylation of TRA2 proteins counters their localization to speckles, thereby modulating interstasis. Thus, the condensation properties of nuclear speckles act as a sensor for interstasis, a collective negative-feedback loop that co-regulates mRNAs of highly dosage-sensitive genes, which primarily encode nuclear condensation-prone proteins.

## Abstract (web summary)

The authors discover a homeostatic process termed interstasis, in which an increased concentration of proteins within RNA–protein condensates induces the sequestration of their own mRNAs.

## Main

Fitness screens in multiple eukaryotic species have shown that proteins containing intrinsically disordered regions (IDRs) are more likely to be toxic when their dosage is increased^1^. The toxicity induced by the increased dosage of individual IDR-containing proteins in yeast has been linked to their propensity to phase separate and form biomolecular condensates^3^. Proteins that are prone to phase separation have the greatest mismatch between protein and RNA abundance, indicating that post-transcriptional mechanisms strongly affect the protein abundance^1,2^. Many proteins can interact with the RNA or DNA of their own gene, yet no sensors or effectors are known that could regulate the collective expression of condensation-prone proteins^5–8^.

Co-condensation of proteins is partly determined by the molecular grammar of short motifs and biophysically similar amino acids in their IDRs^9–12^. Many IDRs contain repetitive amino acid arrangements and are thus classified as low-complexity domains (LCDs). One type of LCD is the R-MCD, where arginines are interspersed among other positively and negatively charged amino acids. R-MCDs promote localization of proteins into nuclear speckles^10^. Major overexpression of artificial R-MCDs has been shown to increase speckle cohesion to the extent that all polyadenylated mRNAs can become retained in the speckles, highlighting the need for homeostatic co-regulation of such proteins^10^.

## LCDs are encoded by multivalent RNA

**Fig. 1:**
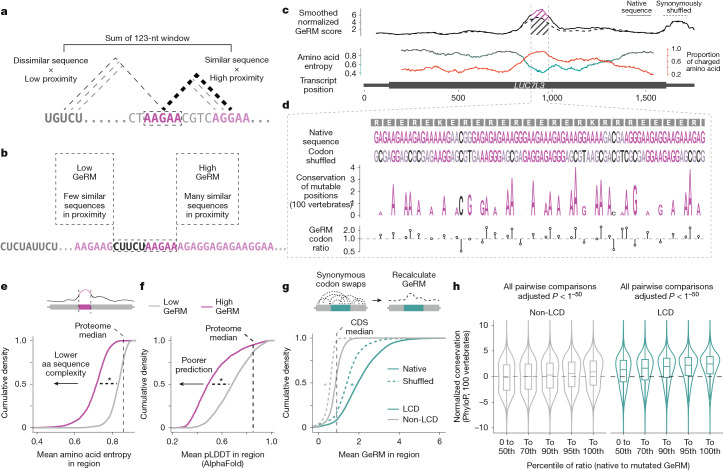
Generalized RNA multivalency scoring identifies codon-biased multivalent regions that encode low-complexity domains. **a**, Calculation of a GeRM score for an individual 5-mer. **b**, An example of two 5-mers with either high or low GeRM scores. **c**, An example transcript from the gene *LUC7L3*. The smoothed GeRM score is shown at the top (solid line), and the dashed line shows the average smoothed GeRM score after synonymous codon shuffling. The amino acid entropy of the encoded sequence is shown at the bottom (black and teal line), and the proportion of charged amino acids in that window is shown as the orange line. **d**, The native DNA and amino acid sequences of *LUC7L3* within the GeRM peak and a synonymously codon-shuffled sequence. Conservation across 100 vertebrates (PhyloP) for each position that can tolerate synonymous mutation is shown by the height of the letter. Below, a ratio of GeRM scores for the native codon choice to average scores of any synonymous mutation is also shown. **e**, The mean entropy for amino acid sequences encoded inside high GeRM CDS regions (black line) outside but within the same protein (grey line). **f**, As in **e**, but comparing the AlphaFold-predicted pLDDT values inside (black line) and outside (grey line) protein regions encoded by high GeRM regions. **g**, The mean GeRM scores within CDS regions encoding LCDs (black lines) or the rest of the CDS (grey lines). The mean GeRM scores in those regions after synonymously reshuffling the codons across the transcriptome (dashed lines) are also shown. **h**, The normalized conservation across 100 vertebrates of synonymously mutable positions in coding sequences that either encode LCDs or do not. Codons are binned by the degree that the native codon choice supports sequence multivalency, in which codons with the highest ratio support the multivalency the most. Unless otherwise stated, all pairwise significance testing were performed using FDR-corrected Welch *t*-tests, where *P < 1^−15^. Precise *P* values can be found in the Source Data. The boxplots show the median (centre), upper and lower quartiles (hinges), and the nearest value within 1.5 times the interquartile range from the quartile (whiskers). Source data

Biophysically similar amino acids tend to have similar codons, a characteristic of genetic code that is thought to have emerged to mitigate the effects of non-synonymous point mutations or translation errors^13,14^. We asked whether the mRNA regions that encode LCDs contain repetitive arrangements of similar sequences, termed ‘multivalent regions’. To generalize the analysis of multivalent RNA sequences beyond specific motif sets, we designed a scoring algorithm that assigns a generalized RNA multivalency (GeRM) score for each nucleotide within provided RNA sequences (Fig. 1a). For a given *k*-mer at each position along a transcript, a GeRM score is calculated by assessing the similarity of surrounding *k*-mers, weighted based on the proximity between *k*-mers (Fig. 1a). A high GeRM score thus indicates that the *k*-mer is present in highly multivalent RNA regions, where large numbers of similar *k*-mers are found in close proximity (Fig. 1b). We applied the GeRM algorithm to the longest coding transcript isoform for every human gene and then focused our analyses on the coding sequences (CDSs). For example, the profile of *LUC7L3* mRNA reveals a region of high GeRM scores (‘GeRM region’) that is highly GA rich and that encodes exclusively charged amino acids (Fig. 1c,d).

We first systematically defined the GeRM regions that were above the threshold of the 98th percentile of the smoothed GeRM scores. To assess the complexity of amino acid sequences in GeRM regions, we calculated their information entropy using a sliding window and found that their entropy was significantly lower than the remaining sequences of the same proteins and to the average throughout the proteome, demonstrating that GeRM regions commonly encode LCDs (Fig. 1e). Moreover, we used AlphaFold2 prediction confidence to approximate protein disorder and found that GeRM regions preferentially encode IDRs, as they are not predicted to be structured (Fig. 1f).

## Codon biases promote RNA multivalency

**Extended Data Fig. 1:**
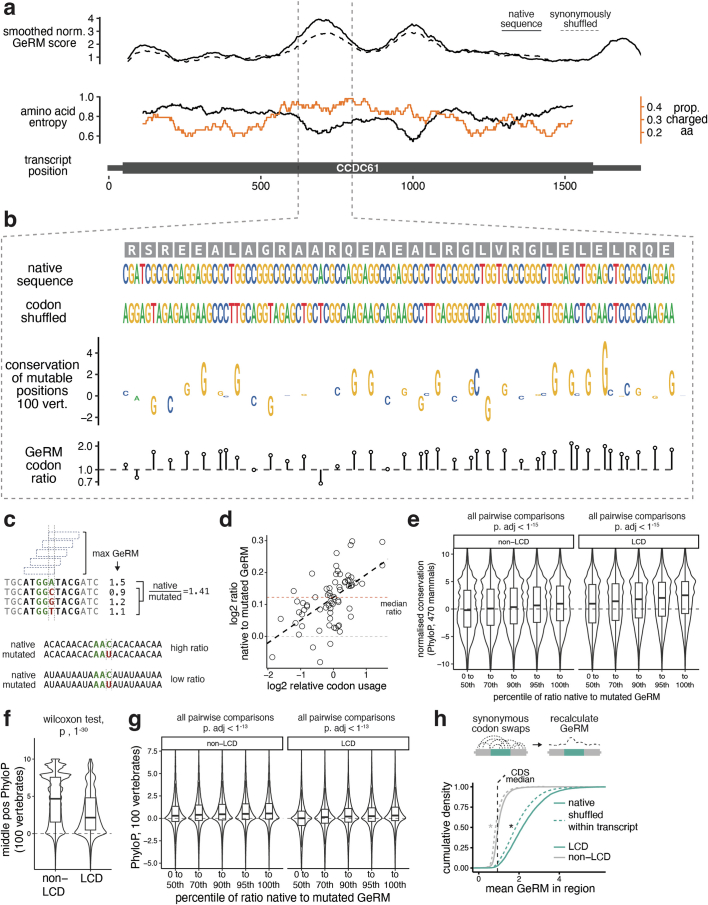
Conservation of multivalency-promoting codon biases. a, An example of a transcript from the gene CCDC61. The smoothed GeRM score is shown along the transcript in the upper panel (solid line), with the dashed line depicting the average smoothed GeRM score after synonymous codon shuffling. The amino acid entropy of the encoded sequence is shown in the lower panel (black line), while the proportion of charged amino acids in that window is shown in the orange line. b, The native DNA and amino acid sequences of CCDC61 within the GeRM peak. A codon shuffled sequence in which all codons must be synonymously shifted is shown. The conservation across 100 vertebrates (PhyloP) for each position that can tolerate synonymous mutation is shown, with the height of the letter corresponding to the PhyloP score. Below, a ratio is shown that compares the GeRM scores associated with the native codon choice to the GeRM scores associated with any possible synonymous mutation. c, a schematic detailing the calculation of the ratio of GeRM between native codon choices and synonymous mutations. d, the mean log2-transformed ratio for each type of possible synonymous mutation. For example, for the arginine codon CGG, position 1 is considered a synonymously mutable position (CGG -> AGG) and position 3 is considered separately as a mutable position (e.g CGG -> CGC). Relative codon usage is calculated by scaling the transcriptomic codon usage such that the mean is 0 and the standard deviation is 1. e, The normalised conservation across 470 mammals of synonymously mutable positions in coding sequences that either encode LCDs or do not. Codons are binned by the degree that the native codon choice supports sequence multivalency, where codons with the highest ratio support the multivalency the most. Conservation values are normalised to the middle position of the codon, which is never synonymously mutable, and each codon is normalised to have a median conservation value of 0. f, PhyloP scores across 100 vertebrates of the middle positions of codons inside or outside of LCDs. g, As in Fig. 1h and Extended Data Fig. 1e, but using unnormalised PhyloP scores across 100 vertebrates. h, The mean GeRM scores within CDS regions encoding LCDs (black lines), or the rest of the CDS (grey lines). The mean GeRM scores in those regions after synonymously reordering the codons within each transcript (dashed lines) Unless otherwise stated, pairwise significance testing are FDR-corrected Welch t-tests, where * = p < 1^−15^. Precise p-values are found in Source Data. Source data

If RNA multivalency was functionally important, one would expect a selection pressure for biases in codon usage that reinforce the multivalency of LCD-encoding regions. To examine such biases, we synonymously shuffled the codons within each transcript and calculated the mean GeRM potential across ten shuffles (Fig. 1c,g). In the example of *LUC7L3*, codon choices reinforce the GA richness of the CDS region that encodes an R-MCD and is almost entirely composed of charged amino acids (Fig. 1c,d). Conversely, in the example of *CCDC61*, codon choices reinforce the G richness of the CDS region that encodes an LCD that contains only approximately 40% of charged amino acids and is rich in arginines, alanines and glycines (Extended Data Fig. 1a,b).

To more broadly assess how codon biases reinforce the multivalency of LCD-encoding CDS regions, we then defined LCDs as regions in the bottom 2% of amino acid entropy (Supplementary Table 7). The GeRM potential in regions encoding LCDs was markedly higher than in the rest of the CDS, but synonymously shuffling the codons either across the transcriptome or within each transcript significantly reduced the GeRM potential (Fig. 1g and Extended Data Fig. 1h). This suggests that, especially in regions encoding LCDs, codon choices tend to promote the multivalency potential of the RNA.

We speculated that if codon usage supports high multivalency of sequences encoding LCDs, then these codon usage biases would be evolutionarily conserved. We tested every possible synonymous codon substitution in the transcriptome and calculated the change in GeRM potential of each overlapping 5-mer (Extended Data Fig. 1c). Next, we compared the GeRM potentials of the native *k*-mers and the mutated *k*-mers. When the ratio of native to mutated GeRM potential is high, then the native codon usage promotes the local multivalency potential. The average ratio for all but four possible synonymously mutable codons was positive, with more common codons having higher ratios on average (Extended Data Fig. 1d). To account for the general differences in amino acid composition and conservation of LCDs (Extended Data Fig. 1f), we normalized the conservation of each synonymously mutable codon and then compared the conservation of the synonymously mutable nucleotide (typically the wobble position) to the conservation of the middle nucleotide in each codon, which can never be synonymously mutated. We found that the more strongly a native codon promotes multivalency, the more conserved the codon is across mammals or vertebrates, and that this effect is especially strong within the regions that encode LCDs (Fig. 1h and Extended Data Fig. 1e,g). This analysis suggests that evolutionary selection pressure tends to preserve codons that enhance high multivalency potential of RNA sequences encoding LCDs.

## CDS multivalency relates to LCD classes

**Fig. 2:**
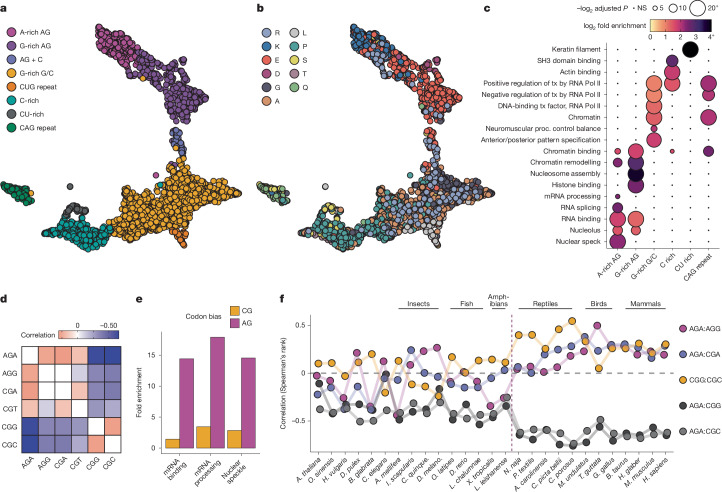
GeRM subtypes encode LCDs with distinct properties and functions. **a**, UMAP embedding in which each point is a high GeRM CDS region and is assigned to a cluster. **b**, UMAP embedding from **a**, with each GeRM region recoloured to show the most common amino acid encoded within the region. **c**, Heatmap of Gene Ontology fold enrichments (colour) and significance values (size) for genes containing GeRM regions from the clusters shown in **a**. NS, not significant; proc., process; tx, transcription. **d**, The correlation (Spearman’s rank) of arginine codon usage within LCDs containing at least 20% arginine (R-LCDs). **e**, The Gene Ontology fold enrichments for genes containing R-LCDs encoded by GA-rich codons or CG-rich codons. **f**, Analysis of codon usage correlations within R-LCDs in different species. Species to the right of the dashed vertical line are amniotes. The black dashed line denotes no correlation.

**Extended Data Fig. 2:**
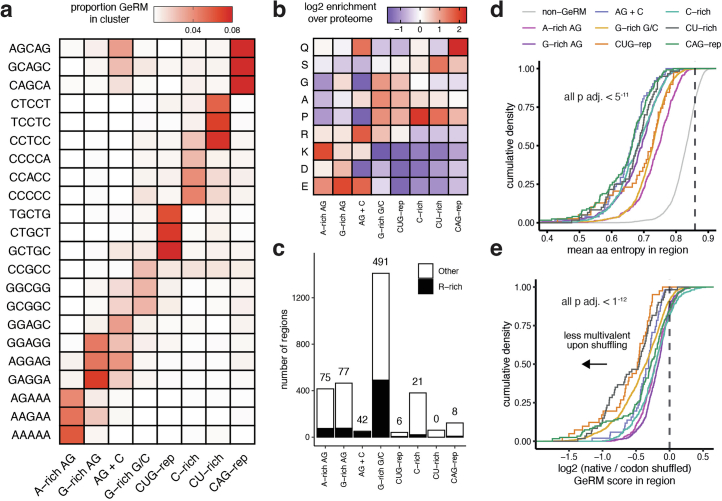
Clusters of multivalent coding sequences and their coding biases. a, The contribution to the total GeRM score in each GeRM cluster that is accounted for by a given 5-mer. Only the three most common 5-mers per cluster are shown. b, The relative occurrence of amino acids encoded by different GeRM regions relative to their occurrence in the entire proteome. Only amino acids with notable enrichments are shown. c, The number of GeRM regions per cluster encoding at least two-fold more arginines than expected based on occurrence in the proteome. d, The mean amino acid entropy calculated in sliding windows for amino acids encoded by different GeRM regions or the amino acid sequences encoded by the rest of the CDS (grey line). The dashed line represents the mean amino acid entropy for the entire proteome. Statistical comparisons are Welch t-tests between the GeRM regions and the non-GeRM regions from the same set of proteins, with FDR correction. e, The log2-transformed ratio between the total GeRM calculated from the native sequence within a GeRM region and the mean total GeRM score in the same region when codons have been synonymously shuffled five times within each transcript. Statistical tests are one sample t-tests, with FDR correction.

To understand whether RNA multivalency could serve as a template for co-regulation of co-condensing proteins, we assessed its relationships to the LCD molecular grammar. We calculated the contribution of each 5-mer to the total GeRM score of each region, reduced the dimensionality of the data with UMAP and clustered all protein-coding GeRM regions using HDBSCAN. We observed diverse clusters, some of which represented trinucleotide repeats, whereas the largest clusters lacked stereotyped repetition: a GC-rich cluster, a C-rich cluster and three GA-rich clusters (one cluster with more adenines, one with more guanines and one with interspersed cytosines; Fig. 2a, Extended Data Fig. 2a and Supplementary Table 6). All types of GeRM regions encoded LCDs (Extended Data Fig. 2d), and GeRM regions with similar multivalent RNA motifs tend to encode LCDs that are dominated by similar amino acids (Fig. 2b and Extended Data Fig. 2b). The GA-rich GeRM regions encoded domains rich in charged amino acids, with the ratio of adenine to guanine in the multivalent motifs biasing the encoded domain towards positive or negative charge, respectively.

Next, we asked whether each type of GeRM region is enriched in mRNAs that encode proteins with related functions. We performed an ontology analysis of gene sets containing each type of GeRM region and found that each set was associated with specific terms (Fig. 2c). The GA-rich GeRM regions were primarily associated with nuclear RNA regulation or chromatin-related terms, whereas GC-rich and CAG-repeat GeRM regions were enriched mainly for transcription-related terms, which agrees with previous studies showing GC bias for codons in transcription factors^15^. The C-rich GeRM regions were most enriched in the SH3 domain and actin-binding terms, whereas the CU-rich regions were enriched for keratin filament genes. Therefore, each type of RNA multivalency in CDS regions is linked to specific classes of LCDs and enriched in functionally related groups of transcripts.

## R-MCDs have distinct codon biases

**Extended Data Fig. 3:**
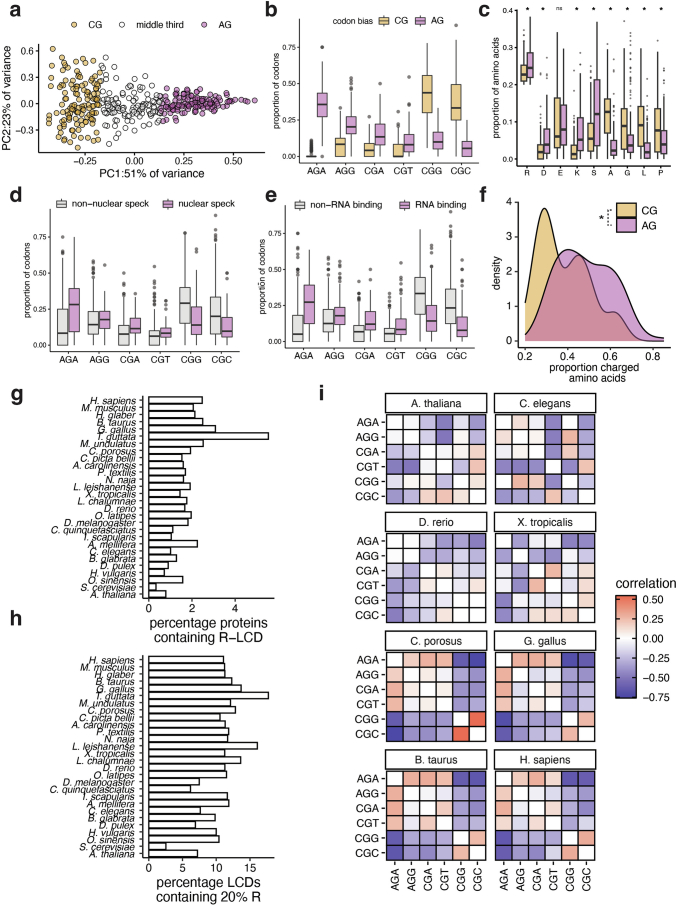
Codon biases within R-LCDs across species. a, Principal component analysis of the arginine codon usage within LCDs containing at least 20% arginine (R-LCDs). R-LCDs are categorised into three groups of equal size using the first principal component. b, The codon usage of the groups of R-LCDs defined in panel a. c, The representation of amino acids within the groups of R-LCDs defined in panel a. d-e, The codon usage of R-LCDs from genes with either ‘nuclear speck’ or ‘RNA binding’ gene ontology terms compared to R-LCDs without those annotations. f, The proportion of charged amino acids within the R-LCD categories defined in panel a. g, The percentage of proteins containing at least one R-LCD in different species. h, the percentage of LCDs that contain at least 20% arginine in different species. i, the Spearman’s rank correlations of codon usage within R-LCDs in different species. Pairwise significance testing are FDR-corrected Welch t-tests, where * = p < 0.05, ** = p < 0.01, *** = p < 0.001. Precise p-values are found in Source Data. Source data

All RNA multivalency classes in coding regions are reinforced by biased codon usage, as evident by significantly decreased RNA multivalency in all types of GeRM regions after codon shuffling (Extended Data Fig. 2e). These biases are driven mainly by codon choice for amino acids that contain a large number of possible codons, such as arginine, which is common in multiple types of LCDs (Fig. 2b and Extended Data Fig. 2c). We calculated the arginine codon usage within each LCD that contained at least 20% arginine (R-LCDs), which could be separated based on codon-usage principal component into those encoded primarily by GA-rich or CG-rich codons (Extended Data Fig. 3a,b). Those with GA-rich arginine codons were highly enriched for other charged residues and thus included R-MCDs (Extended Data Fig. 3c,f), whereas R-LCDs with CG-rich codons contained more proline, alanine and glycine (Extended Data Fig. 3c). Of note, the GA-biased R-LCDs are much more highly enriched for nuclear speckle, mRNA-processing and RNA-binding Gene Ontology terms compared with GC-rich R-LCDs (Fig. 2e). Conversely, mRNAs encoding proteins with nuclear speckle or RNA-binding Gene Ontology terms show a strong bias towards GA-rich arginine codons (Extended Data Fig. 3d,e). Therefore, arginine codon choice within GeRM regions conforms to the multivalent identity of the region by mimicking the sequences of other codons encoding charged amino acids, thereby maximizing the multivalency of RNA regions encoding functionally distinct classes of R-LCD. As representative examples, the arginine codon biases in *LUC7L3* and *CCDC61* R-LCDs reinforce their distinct AG-rich and CG-rich multivalency, respectively (Fig. 1c,d and Extended Data Fig. 1a,b).

As a measure of the distinct arginine codon preference in the two classes of R-LCDs, we observed a positive correlation in R-LCDs between AGA, AGG and CGA arginine codons, and between CGG and CGC codons, and strong anticorrelations between these two groups (Fig. 2d). We used this approach to ask at what point in evolution this codon bias in R-LCDs emerged. We observed the correlations and anticorrelations between AG and CG arginine codons in R-LCDs across a diverse range of species. Although the total proportions of R-LCDs did not dramatically change across species (Extended Data Fig. 3g,h), we observed that the correlations between CG-rich codons, between GA-rich codons and the anticorrelation between these groups were stable from humans to reptiles, but that this pattern was less pronounced in amphibians and other species that diverged earlier in evolutionary history (Fig. 2f and Extended Data Fig. 3i). Thus, the codon biases that reinforce the distinct RNA multivalency of two R-LCD classes have become most pronounced in terrestrial vertebrates.

## R-MCD dosage drives GA-rich mRNA capture

**Fig. 3:**
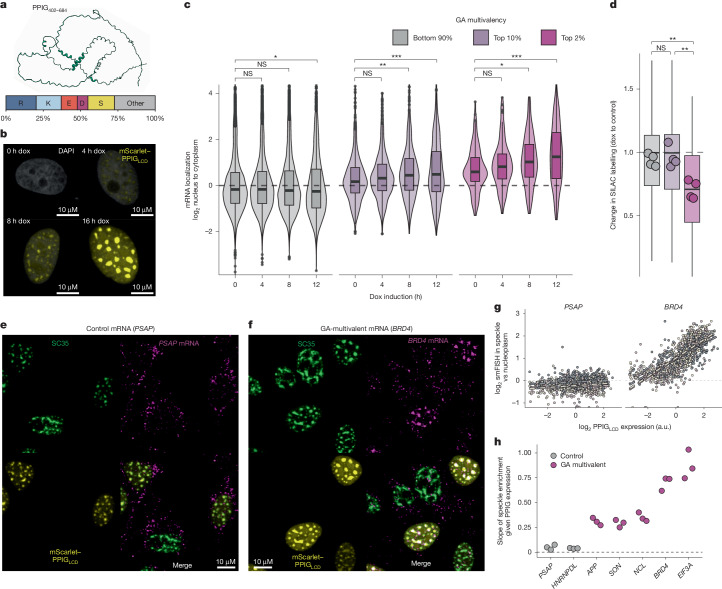
R-MCD expression drives dose-dependent sequestration of GA-rich genes in nuclear speckles. **a**, AlphaFold prediction of the PPIG_LCD_ structure and the representation of amino acids within the domain. **b**, Expression of PPIG_LCD_ over time after doxycycline (dox) induction from a single experiment. **c**, Nuclear–cytoplasmic distribution of mRNAs with different degrees of GA multivalency in response to different timepoints of PPIG_LCD_ expression. *n* = 6,367, 566 and 141 genes for the bottom 90%, top 10% and top 2%, respectively, with calculations performed on values from 4 independent replicates. **d**, The change in proportion of labelled peptides after 8 h of SILAC labelling per protein when inducing PPIG_LCD_ expression for 12 h compared with uninduced cells. The boxplots show the mean change per protein across replicates, and the dot plots show the mean across all proteins for a given replicate (*n* = 4 independent experiments). **e**,**f**, Example images from a set of three independent experiments showing the HCR-FISH signal for *PSAP* (control; **e**) or *BRD4* (GA multivalent; **f**) mRNAs, with SC35 immunofluorescence and mScarlet–PPIG_LCD_. **g**, Quantification of the enrichment of the HCR-FISH signal within nuclear speckles versus the nucleoplasm per nucleus with respect to PPIG_LCD_ expression. Three independent replicates are plotted in different colours, and regression slopes are plotted in dashed lines. **h**, The slopes of linear regression models of the relationship between mScarlet–PPIG_LCD_ expression and mRNA enrichment within the speckle for control and GA-multivalent mRNAs. Pairwise significance testing were performed using FDR-corrected Welch *t*-tests, where *P < 0.05, **P < 0.01 and ***P < 0.001. Precise *P* values can be found in the Source Data. The boxplots show the median (centre), upper and lower quartiles (hinges), and the nearest value within 1.5 times the interquartile range from the quartile (whiskers). Black dashed lines denote no change between conditions or no correlation, as appropriate. Source data

**Extended Data Fig. 4:**
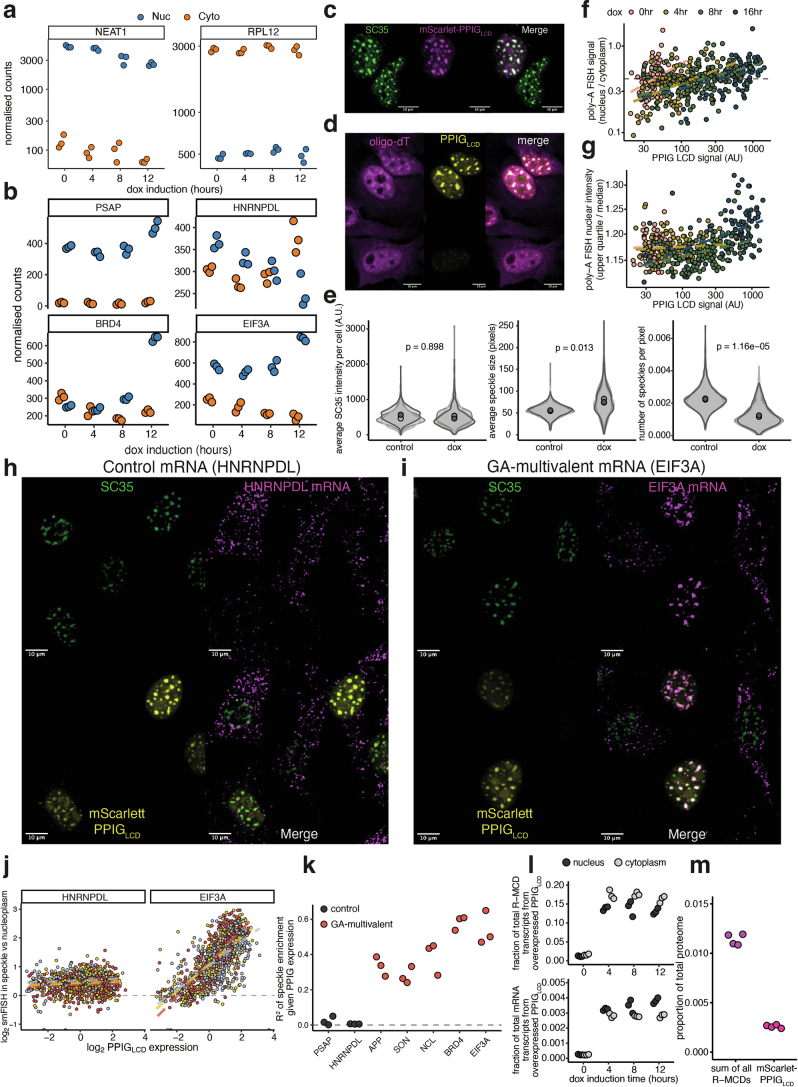
Capture of GA-multivalent mRNAs within nuclear speckles driven by R-MCD concentration. a, Normalised counts for the nuclear lncRNA NEAT1 and the efficiently exported mRNA RPL12 in nuclear and cytoplasmic fractions at different PPIG_LCD_ expression timepoints. b, Normalised counts for control (PSAP, HNRNPDL) and GA-rich (EIF3A, BRD4) RNAs, as in panel a. c, Co-localisation of mScarlet-PPIG_LCD_ with nuclear speckles. d, Oligo-dT FISH of cells expressing mScarlet-PPIG_LCD_. e, Changes in the nuclear SC35 intensity and the size and number of speckles per nucleus after expression of mScarlet-PPIG_LCD_ across 3 replicates, with at least 100 nuclei per replicate. f, The ratio of total oligo-dT FISH signal in the nucleus and cytoplasm per cell compared the the intensity of mScarlet-PPIG_LCD_ in the nucleus. Points are coloured based on the length of expression induction with doxycycline. g, The ratio of the upper quartile to the median of nuclear oligo-dT FISH signal as a proxy for the relative intensity of nuclear speckle signal. Ratios are compared the intensity of mScarlet-PPIG_LCD_ in the nucleus, and points are coloured as in panel f. h-i, Example images showing HCR-FISH signal for HNRNPDL (control) or BRD4 (GA-multivalent) mRNAs, with SC35 immunofluorescence and mScarlet-PPIG_LCD_. j, Quantification of the enrichment of HCR-FSH signal within nuclear speckles versus nucleoplasm per nucleus with respect to PPIG_LCD_ expression. 3 independent replicates are plotted in different colours and regression lines are plotted in dashed lines. k, The fits of linear regression models (R^2^) of the relationship between mScarlet-PPIG_LCD_ expression and mRNA enrichment within the speckle for control and GA-multivalent mRNAs for each of the 3 replicates. l, Quantification of the proportion of total R-MCD encoding transcripts or total mRNAs that are the overexpressed PPIG_LCD_ transcript in the dox induction time course sequencing experiment. m, Quantification of the abundance of all R-MCD proteins or the overexpressed mScarlet-PPIG_LCD_ after 12 h of doxycycline induction.

As the accumulation of R-MCDs affects the properties of nuclear speckles^10^, we investigated whether speckles enable any feedback regulation of R-MCD proteins, and whether GA-rich GeRM regions have any role in such regulation. To assess this, we combined the region encoding the R-MCD C terminus of peptidylprolyl isomerase G (PPIG; Fig. 3a) fused to mScarlet into a doxycycline-inducible PiggyBac vector, which was integrated into a stable polyclonal HeLa cell population. After 16 h of induction, we observed mScarlet–PPIG_LCD_ localization to the nucleus, where it colocalized with SC35, a nuclear speckle marker (Extended Data Fig. 4c). Expression of PPIG_LCD_ resulted in fewer, larger speckles per nucleus, without reducing the intensity of SC35 staining (Extended Data Fig. 4e), consistent with previously reported effects of R-MCDs on speckles^10^.

We first asked whether the expression of the R-MCD had a dose-dependent effect on the nuclear–cytoplasmic localization of endogenous GA-rich mRNAs. We performed 3′ end sequencing on the nuclear and cytoplasmic fractions of our reporter cell line after 0, 4, 8 and 12 h of mScarlet–PPIG_LCD_ induction to assess the effect of its gradual accumulation (Fig. 3b, Extended Data Fig. 4a, Supplementary Fig. 3). We then categorized genes based on their total CDS GA multivalency and looked at the nuclear–cytoplasmic distribution of these genes over time. The nuclear–cytoplasmic distribution of most mRNAs was unaffected by the expression of the reporter construct, but the group of mRNAs with high GA-rich multivalency became increasingly enriched in the nucleus over time, with a quantitative relationship between the degree of GA-rich multivalency and the degree of nuclear retention (Fig. 3c).

To investigate the effect of the observed nuclear retention of GA-rich multivalent transcripts on protein synthesis, we performed pulsed stable isotope labelling by amino acids in cell culture (pSILAC). PPIG_LCD_ expression was induced for 12 h, followed by an 8-h pulse labelling period to selectively label and quantify proteins newly synthesized during the PPIG_LCD_ induction window. Comparison of heavy-to-light peptide ratios between doxycycline-induced and uninduced controls revealed a selective decrease in the proportion of labelled peptides encoded by mRNAs with high GA multivalency (Fig. 3d), with minimal effect observed on the translation of all other proteins. Thus, the nuclear retention of GA-rich multivalent transcripts upon PPIG_LCD_ expression corresponds with downregulated translation of the proteins that these mRNAs encode.

To determine whether GA-rich transcripts are specifically sequestered into nuclear speckles, we designed hybridization chain reaction fluorescence in situ hybridization (HCR-FISH) probes against five GA-rich multivalent mRNAs and two non-multivalent control mRNAs. We then induced PPIG_LCD_ expression for 16 h and performed HCR-FISH along with SC35 immunofluorescence (Fig. 3e,f and Extended Data Fig. 4h,i). We found a dose-dependent increase in the proportion of single-molecule FISH (smFISH) signal in the speckles relative to the nucleoplasm for all five multivalent RNAs, but not for either of the two control mRNAs, as evidenced by the slopes and fits of the regression models (Fig. 3g,h and Extended Data Fig. 4j,k). These observations correspond to changes in nuclear–cytoplasmic distribution in our sequencing experiments (Extended Data Fig. 4b). Under these conditions we also observed a slight dose-dependent enrichment of poly-A^+^ mRNA in nuclear speckles, accompanied by a mild increase in the proportion of nuclear mRNA, indicating that mRNA sequestration is selective (Extended Data Fig. 4d,f,g).

**Extended Data Fig. 5:**
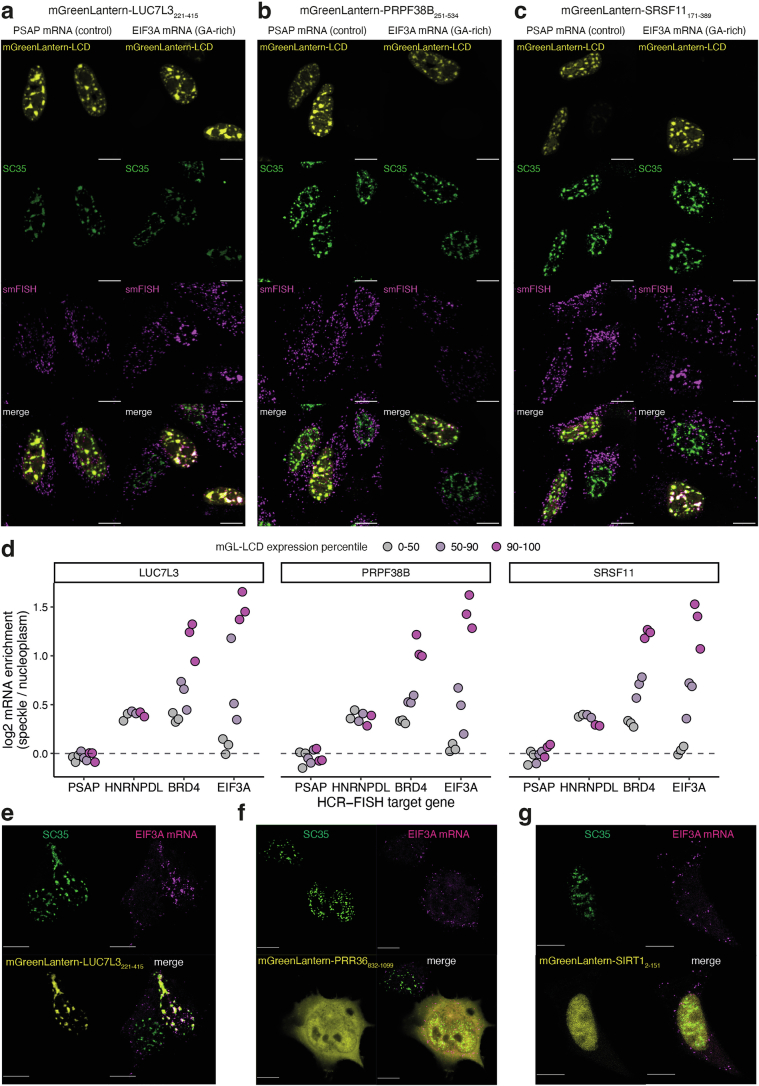
Different R-MCDs, but not other LCDs, drive selective GA-multivalent mRNA retention in nuclear speckles. a-c, Example images showing HCR-FISH signal for PSAP (control) or EIF3A (GA-multivalent) mRNAs, with SC35 immunofluorescence in cells expressing different R-MCDs (LUC7L3_221–415_, PRPF38B_251–534_ and SRSF11_171–389_) fused to mGreenLantern. d, Quantification of enrichment of HCR-FISH signal within nuclear speckles compared to the nucleoplasm for cells expressing varying amounts of the R-MCDs shown in panels a-c. e-g, As in panels a-c, but showing EIF3A HCR-FISH signal for cells expressing either an R-MCD with GA-rich GeRM (LUC7L3_221–415_) or a LCD associated with a different GeRM signature (C-rich GeRM, Pro-rich: mGreenLantern-PRR36_832–1099_; GC-rich GeRM, Ala/Glu/Gly-rich: mGreenLantern-SIRT1_2–151_). Pairwise significance testing are FDR-corrected Welch t-tests, where * = p < 0.05, ** = p < 0.01, *** = p < 0.001. Precise p-values are found in Source Data

We next asked whether this effect is generalizable across R-MCDs. We transfected HeLa cells with mGreenLantern-fused R-MCDs from three additional proteins (LUC7L3, PRPF38B and SRSF11) and repeated HCR-FISH 24 h post-transfection, with two GA-rich multivalent mRNAs and two non-multivalent controls (Extended Data Fig. 5a–c). This induced the selective speckle retention of GA-rich mRNAs, consistent with our observations with PPIG_LCD_ (Extended Data Fig. 5d). We did not observe this effect when transfecting other LCDs with different amino acid identities (Extended Data Fig. 5e–g). We conclude that variations in the concentration of any given R-MCD, or variations in the combined concentrations of all R-MCDs, drive the selective retention of GA-rich mRNAs in nuclear speckles. We quantified PPIG_LCD_ expression at the protein and RNA level and found that a 15% increase in the total abundance of R-MCDs in the cell is sufficient to induce selective mRNA retention (Extended Data Fig. 4i,m). Thus, modest changes in the concentration of R-MCDs can selectively control the export of mRNAs containing GA-rich GeRM regions, which encode R-MCD or other charged proteins.

## Co-regulation of condensation-prone proteins

**Extended Data Fig. 6:**
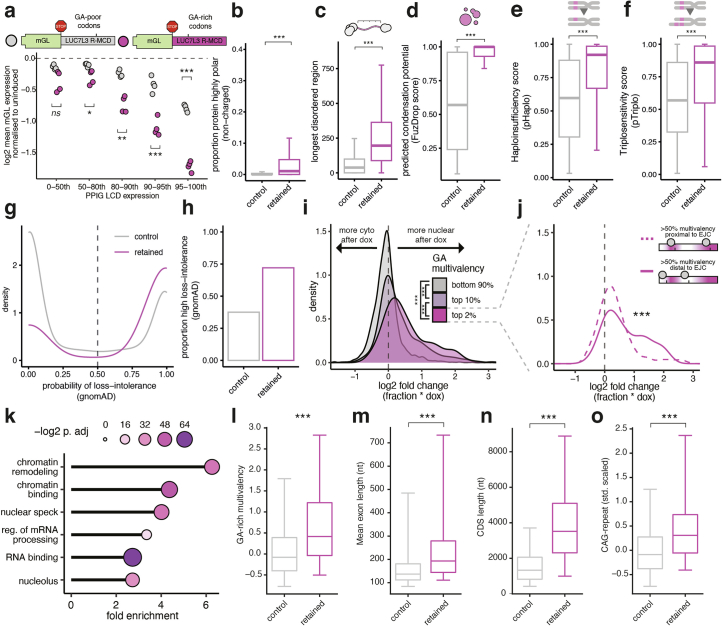
Properties of the mRNAs retained in the nucleus upon PPIG overexpression and the proteins they encode. a, Flow cytometry measurements of mGreenLantern signal from cells transfected with constructs encoding mGreenLantern with a GA-rich or less GA-rich 3’UTR. PPIG_LCD_ was expressed for 16 h. mGreenLantern intensities are normalised to mGreenLantern transfection without PPIG_LCD_ expression. b, The proportion of proteins made up of highly polar (non-charged) regions (regions of 40 amino acids with greater than 40% polar, non-charged residues). Proteins encoded by genes that showed significant nuclear mRNA retention upon expression of PPIG_LCD_ are compared to proteins from expressed genes that did not exhibit retention. c, length of longest predicted disordered region. d, FuzDrop scores for retained and control genes. e, Dosage sensitivity scores (haploinsufficiency) for retained and control genes. f, Dosage sensitivity scores (triplosensitivity) for retained and control genes. g, The distribution of predicted probabilities of loss-intolerance as determined by gnomAD for retained and control genes. h, The proportion of retained or control genes with a gnomAD score greater than 0.5. i, Per mRNA interaction effects describing the change in nuclear-cytoplasmic distribution after 12 h of PPIG_LCD_ expression for mRNAs with different degrees of GA multivalency. j, Interaction effects from panel b for mRNAs in the top 2% of GA multivalency. mRNAs are classified based on whether 50% of the total GA-multivalency occurs within 50 nt of a predicted exon junction complex site. k, Selected Gene Ontology enrichments for retained genes over all expressed genes. l, GA-multivalency for retained and control genes. m, Mean exon length for retained and control genes. n, CDS length for retained and control genes. o, CAG-multivalency for retained and control genes. Pairwise significance testing are FDR-corrected Welch t-tests, where * = p < 0.05, ** = p < 0.01, *** = p < 0.001. Precise p-values are found in Source Data. Source data

To assess whether the nuclear retention of GA-rich mRNAs decreases the synthesis of encoded proteins, we created codon-biased versions of a GA-rich region in *LUC7L3* mRNA that encodes an R-MCD (Fig. 1c,d), such that it either had a high or low GA multivalency but equivalent GC content. We then cloned this region as a 3′ untranslated region (UTR) sequence downstream of a CDS encoding mGreenLantern. We transfected each of these two constructs into our mScarlet–PPIG_LCD_ reporter cell line, induced PPIG_LCD_ expression for 16 h, and measured the intensity of mScarlet and mGreenLantern in each cell. We found that as the expression of mScarlet–PPIG_LCD_ increased, expression of mGreenLantern strongly decreased when the 3′ UTR of mGreenLantern contained a highly multivalent GA-rich sequence, whereas the effect was significantly weaker when the sequence was codon biased to decrease the GA content (Extended Data Fig. 6a). Thus, modestly increased concentration of an R-MCD protein decreases the synthesis of other R-MCD proteins due to the broad effect on the retention of endogenous GA-rich mRNAs. We refer to this mutual homeostatic co-regulation of LCD-containing proteins as interstasis.

**Fig. 4:**
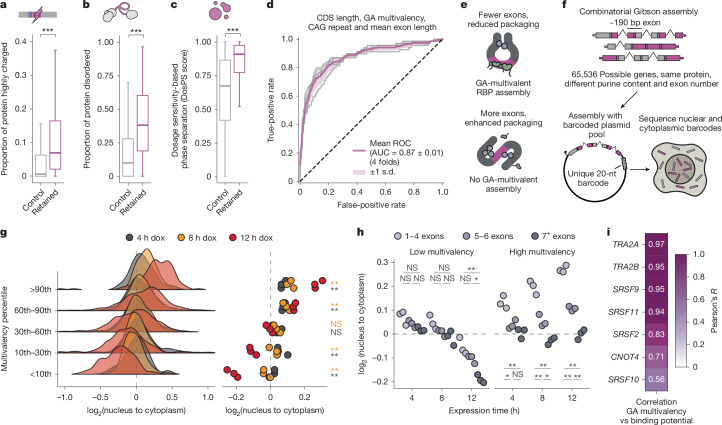
GA multivalency and exon architecture drive selective nuclear retention of mRNAs. **a**, The proportion of proteins made up of highly charged regions (regions of 40 amino acids with greater than 40% charged residues). Proteins encoded by genes that showed significant nuclear mRNA retention upon expression of PPIG_LCD_ are compared with proteins from expressed genes that did not exhibit retention (*n* = 6,543 and 531, respectively). **b**, Proportions of the same proteins as in **a** predicted to be disordered by AlphaFold2. **c**, DosPS scores for the same proteins as in **a**. **d**, ROC curve (purple) indicates the mean performance of the balanced random forest model, trained on the indicated features, across fourfold cross-validation (grey curves) in distinguishing between nuclear-retained mRNAs and mRNAs with unchanged nuclear:cytoplasmic ratios (Methods). The shaded area represents the standard deviation across the fourfolds. **e**, Schematic illustrating how mRNP packaging effects driven by exon length could influence assembly of interstasis-promoting RBPs. **f**, Schematic describing the assembly of the reporter library. **g**, The nuclear:cytoplasmic abundance ratio of reporter transcripts depending on their sequence multivalency and different lengths of expression induction via doxycycline. The distribution of ratios is shown via a ridgeline plot, whereas the individual group means for each replicate are shown as a dot plot (*n* = 3 independent replicates). **h**, The nuclear:cytoplasmic abundance ratio of reporter transcripts over time depending on the number of exons and their multivalency (*n* = 3 independent replicates). **i**, Pearson’s correlations of the multivalency of reporter gene sequences with the binding scores of different RBPs. All pairwise statistical comparisons were performed using a Welch *t*-test with FDR correction for multiple testing, where *P < 0.05, **P < 0.01 and ***P < 0.001. Precise *P* values can be found in the Source Data. The boxplots show the median (centre), the upper and lower quartiles (hinges), and the nearest value within 1.5 times the interquartile range from the quartile (whiskers). Black dashed lines represent no change between conditions. Source data

To further characterize the proteins that are co-regulated through interstasis, we examined the properties of the proteins encoded by mRNAs that were significantly retained in the nucleus after PPIG_LCD_ expression. These proteins are enriched in IDRs, which are longer than average and have increased densities of charged amino acids (Fig. 4a,b and Extended Data Fig. 4c) and to a lesser extent also non-charged polar amino acids (Extended Data Fig. 6b). Moreover, their genes are enriched for ontology terms related to the nuclear speckle, chromatin remodelling, RNA binding and RNA processing (Extended Data Fig. 6k). To account for the strong enrichment of Gene Ontology terms related to nuclear compartments, we compared all proteins that localize to speckles, nucleoplasm or nuclear bodies as defined by the Human Protein Atlas^16^ to the subset of these proteins that are co-regulated through interstasis, and found that these have extremely high condensation potential as predicted by the FuzDrop model^17^ (Extended Data Fig. 6d).

Genes encoding IDR-containing proteins were reported to be more dosage sensitive in yeast, *Drosophila melanogaster* and *Caenorhabditis elegans*^1^. Therefore, we analysed the dosage sensitivity that was estimated from the analysis of rare copy-number variants in the human genome^18^. This identified a striking trend for bidirectional dosage sensitivity of the genes encoding nuclear proteins that are under interstatic co-regulation, with high pHaplo and pTriplo scores that correspond to haploinsufficiency and triplosensitivity (Extended Data Fig. 6e,f). Furthermore, bidirectional dosage sensitivity was confirmed by gnomAD scores, showing higher mutational constraint of genes under interstatic regulation than controls^19^ (Extended Data Fig. 6g,h). Thus, interstasis enables homeostatic dosage co-regulation of genes that are bidirectionally dosage sensitive and mutationally constrained. Finally, the genes under interstatic co-regulation had higher scores by the dosage sensitivity-based phase separation predictor model (DosPS)^20^, confirming the combined trends for strong dosage sensitivity and the high condensation potential of their proteins (Fig. 4c). We thus propose that interstasis protects from the toxicity that can arise from deregulation of the most dosage-sensitive genes through its collective homeostatic regulation of the most condensation-prone proteins.

## Exon density counteracts GA multivalency

The packaging and compaction of mRNPs have been recognized as important steps in mRNA maturation and export^21,22^. When an intron is removed from pre-mRNA, the spliceosome deposits the exon junction complex (EJC) upstream of the exon–exon junction. The EJC promotes the packaging of the mRNA into a compact mRNP, facilitating the loading of further mRNA export factors^22^. Previous work has shown that both mRNA sequence and gene architecture can influence mRNA export, but the relationship between sequence-specific RBP assemblies and mRNP packaging remains incompletely understood^23–25^. We reasoned that EJC deposition upstream of exon–exon junctions could limit the accessibility of multivalent sequences, preventing interstatic regulation. To assess this, we trained a random forest classifier to predict transcripts that become retained in the nucleus after 12 h of PPIG_LCD_ expression. The input features of the model included total CDS length, mean exon length (inversely proportional to EJC density) and multivalent RNA features. We trained an array of models using a stepwise feature-selection approach to find that a combination of four features — CDS length, mean exon length, GA-rich and CAG-rich multivalency — led to a model with the best predictive performance (area under the receiver operating characteristic curve (AUROC) = 0.87; Fig. 4d). All of these features individually contributed to mRNA responsiveness; CDS length (AUROC = 0.83) was the strongest determinant of responsiveness, whereas GA-rich and CAG-rich multivalency, as well as mean exon length, further improved the performance of the model (Extended Data Fig. 6l–o). Furthermore, when we categorized mRNAs based on whether 50% of their GA-rich multivalency was within 50 nt of the expected EJC-binding region, GA-rich multivalent sequences were less capable of inducing nuclear mRNA retention upon PPIG_LCD_ overexpression when they were proximal to the EJC (Extended Data Fig. 6i,j).

To address the importance of EJC density and sequence multivalency in the absence of variable CDS length, we designed a novel reporter system in which mScarlet–PPIG_LCD_ is encoded with variable codon choices and variable exon number, whereas its CDS length remains constant. This allowed us to assess whether EJC density could limit the accessibility of multivalent RNA for RBP assembly (Fig. 4e). The reporter plasmid pool was generated by Gibson assembly of eight fragments in sequence, each of which varied in two ways: having GA-rich or GA-poor codons, and either containing or lacking an intron, in which intron splicing efficiency was maximized using in silico mutagenesis to maximize SpliceAI scores^26^. The combination of these two variables leads to four possible options for each fragment of the CDS (Fig. 4f). This generated a library of reporter sequences that all produced the same protein but differed at the RNA level in their degree of GA-rich multivalency and in the number of constitutive introns (Fig. 4f). As previous work has implicated transcript GC content in the steady-state nuclear–cytoplasmic abundances, we ensured that the GC content of the CDS was kept constant^25^. Owing to the constraints on GC content and splicing efficiency, the most highly multivalent sequence was marginally less multivalent than the native RNA sequence that encodes PPIG_LCD_, whereas the least multivalent sequence was still more multivalent than most random CDSs of the same length (Supplementary Fig. 2b).

**Extended Data Fig. 7:**
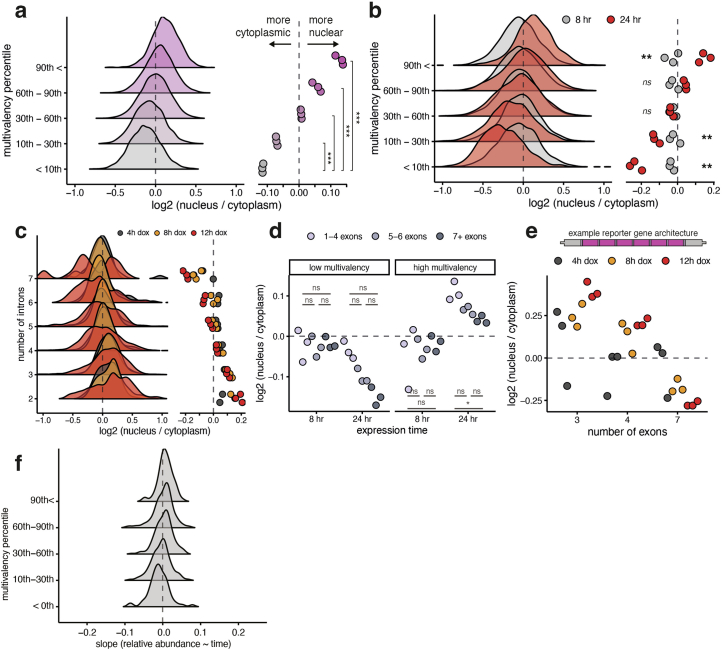
Factors influencing nuclear retention of reporter RNAs. a, The nuclear-cytoplasmic distribution of reporter transcripts binned by the number of introns in their reporter gene. Data from cells transfected with the reporter plasmid pool for 16 h. b, Nuclear-cytoplasmic distributions of reporter transcripts, but comparing cells transfected for either 8 (short) or 24 h (long) with the plasmid pool. c, The nuclear-cytoplasmic distribution of reporter transcripts binned by the number of introns in their reporter gene. Data from cells in which the reporter library is stably integrated and expressed for either 4, 8 or 12 h by the addition of doxycycline. d, The nuclear-cytoplasmic abundance ratio of reporter transcripts over time depending on the number of exons and their multivalency. Data is taken from cells transfected for either 8 (short) or 24 h (long) with the plasmid pool. e, The nuclear-cytoplasmic distribution of reporter transcripts with the same spliced RNA sequence (depicted above the plot) but different number of exons. Data from cells in which the reporter library is stably integrated and expressed for either 4, 8 or 12 h by the addition of doxycycline. f, The slopes from regression models of relative reporter transcript abundance over time were calculated by expressing the reporter pool for 16 h, then collecting fractions at 0, 2, 4 and 8 h after withdrawing dox. A positive slope indicates that the transcript is relatively more stable in the pool, while a negative slope indicates that the transcript is relatively less stable. Pairwise significance testing are FDR-corrected Welch t-tests, where * = p < 0.05, ** = p < 0.01, *** = p < 0.001. Precise p-values are found in Source Data. Source data

Gibson assembly yielded a pool of approximately 45,000 uniquely barcoded plasmids as identified by targeted sequencing (Supplementary Fig. 2c), with a comparable representation of all possible fragments (Supplementary Fig. 2a). Sixteen hours after transfecting this plasmid pool, we isolated the RNA and confirmed with long-read sequencing that all the introns in the reporter were efficiently spliced (Supplementary Fig. 2d–f). Given the roles of nuclear speckles in enhancing splicing efficiency^27^, this enabled us to rule out inefficient splicing as a confounding factor influencing RNA localization. Next, we collected RNA from nuclear and cytoplasmic fractions at 16 h post-transfection and performed targeted sequencing of the plasmid barcodes. Of note, the nuclear retention of reporter mRNAs scaled with the degree of GA-rich multivalency (Extended Data Fig. 7a). To test whether the degree of nuclear retention depends on the extent of the R-MCD expression, we generated a stable inducible pooled cell line expressing the library of reporter constructs, induced expression for 4, 8 and 12 h, and sequenced the nuclear and cytoplasmic barcodes at each timepoint. The nuclear retention of highly multivalent sequences was significantly stronger at 12 h than at 4 or 8 h (Fig. 4g). We replicated this effect by transfecting the reporter plasmid pool for 8 or 24 h and sequencing the nuclear and cytoplasmic barcodes (Extended Data Fig. 7b). To assess the stability of the reporter transcripts, we induced the pool for 16 h, sequenced the RNA after 0, 2, 4 and 8 h, and found no relationship between the multivalency of transcripts and their stability (Extended Data Fig. 7f). Therefore, the expression level of the R-MCD dictates the selective nuclear retention of its own multivalent mRNAs.

We also addressed the role of splicing-mediated EJC loading with our reporter system, because in the reporter pool each of the eight fragments had a chance to contain an intron, thus the total number of exons varied between reporters. If all 8 fragments included an intron, there were 9 exons between 162 and 211 nt in length, whereas most reporters had 5–6 exons. Exon number alone had a weak influence on the nuclear–cytoplasmic distribution of reporter transcripts (Extended Data Fig. 7c). However, for the more multivalent reporter transcripts, exon content had a strong effect on nuclear retention: if a reporter transcript had a small number of longer exons, it was increasingly retained over time, but if a reporter had a greater number of shorter exons, it did not accumulate in the nucleus (Fig. 4h and Extended Data Fig. 7e). We observed the same effect, although weaker, when transfecting the plasmid pool, suggesting that chromatin context may promote the EJC-dependent mRNP packaging (Extended Data Fig. 7d). This confirms that multivalent GA-rich RNA sequences confer the greatest potential for nuclear retention when they are positioned inside long exons, where multivalent RBP assemblies are less able to be influenced by EJC-mediated mRNP packaging.

## TRA2 proteins mediate interstasis

Previous insights from the RNA binding of TDP-43 demonstrated that its condensation promotes binding to highly multivalent RNA regions, which includes binding to its own GU-multivalent mRNA that promotes homeostatic control of TDP-43 dosage^28^. Here we asked whether increased condensation of nuclear speckles could promote the capacity of specific RBPs to recruit GA-multivalent mRNAs into speckles and thus enable interstasis. To identify the RBPs that might bind to GA-multivalent mRNAs in interstasis, we first investigated how the GA multivalency of reporter constructs correlates with the abundance of high-affinity binding motifs for different RBPs. We obtained significantly enriched 5-mers for 79 RBPs (*P* < 0.05) from RNA-Bind-N-Seq experiments^29–31^ and used the abundance of these 5-mers and their *Z* scores to assign a binding potential score to each reporter sequence. We found that GA multivalency had the strongest correlation with the binding potential scores of nuclear speckle proteins TRA2A (*r* = 0.97) and TRA2B (*r* = 0.95; Fig. 4i). Thus, the synonymous mutations of arginine codons are expected to promote binding of TRA2 proteins to the GA-rich multivalent regions, which could mediate the variable nuclear retention of the reporter library.

**Fig. 5:**
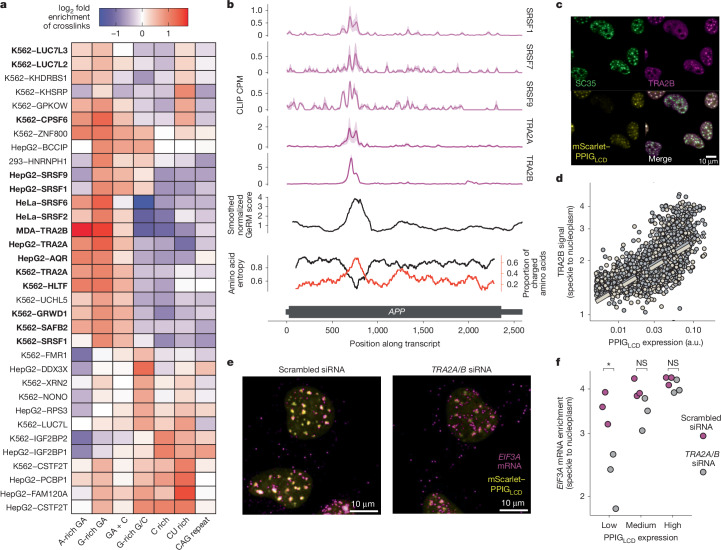
TRA2 proteins are effectors that relocalize multivalent GA-rich mRNAs to nuclear speckles. **a**, Heatmap presenting the CLIP data for RBPs with the greatest preference for multivalent sequences, showing fold enrichment for crosslinks falling within the listed GeRM CDS regions compared with the rest of the same transcript. Datasets in bold are from proteins with known nuclear speckle localization. **b**, Example CLIP crosslinking profiles for SR proteins across an APP transcript, with the smoothed GeRM score, amino acid entropy and proportion of charged amino acids. The solid lines represent the mean across two samples, whereas the shaded regions represent the standard error. CPM, counts per million. **c**, Example TRA2B and SC35 immunofluorescence images from two independent experiments showing nuclei containing variable amounts of mScarlet–PPIG_LCD_. **d**, Quantification of the enrichment of the TRA2B signal within nuclear speckles (based on the SC35 signal) versus the nucleoplasm per nucleus with respect to PPIG_LCD_ expression. Two independent replicates are plotted in different colours, and the regression lines are plotted in dashed lines. a.u., arbitrary units. **e**, *EIF3A* HCR-FISH in nuclei expressing mScarlet–PPIG_LCD_ and treated with either scrambled siRNA or *TRA2A*-targeting and *TRA2B*-targeting siRNA for 48 h (three independent replicates). Scale bars, 10 µm. **f**, Quantification of the enrichment of the *EIF3A* HCR-FISH signal in the nuclear speckle over the nucleoplasm for cells expressing variable amounts of PPIG_LCD_ and treated with either scrambled siRNA or *TRA2A*-targeting and *TRA2B*-targeting siRNA for 48 h. All pairwise statistical comparisons were performed using a Welch *t*-test with FDR correction for multiple testing, where *P < 0.05. Precise *P* values can be found in the Source Data. Source data

**Extended Data Fig. 8:**
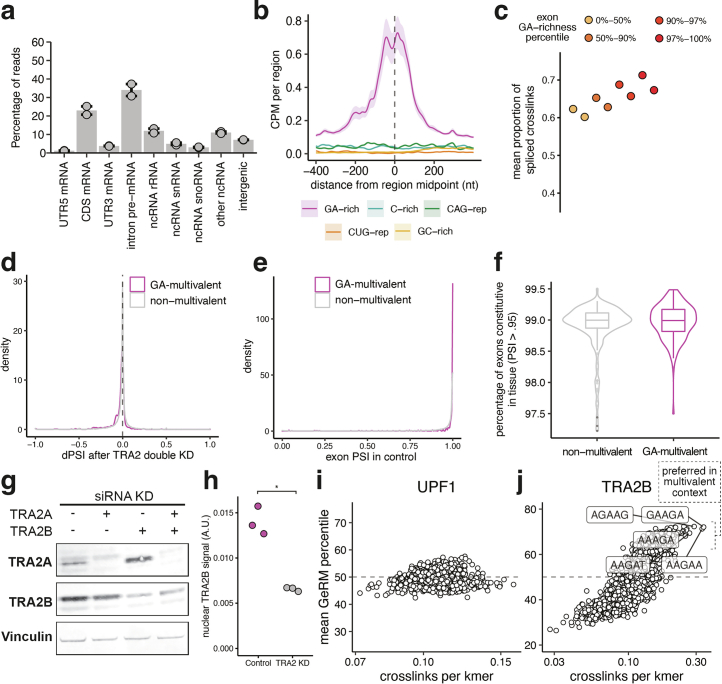
The role of TRA2 proteins assembling on GA-multivalent sequences. a, proportion of mapped Tra2b iCLIP crosslinks falling in different genomic regions. b, a metaprofile of Tra2b iCLIP crosslinks plotted around the midpoints of different types of high GeRM region. Solid lines denote the mean of two replicates, while shaded regions denote the standard error. c, Taking a window of 50 nt from the 3’ end of every internal exon of the longest coding transcript per gene, the proportion of iCLIP reads that contain a splice junction. Exons are binned based on the percentile of their density of GA-containing 5-mers. d, Changes in exon proportion spliced in (dPSI) after double knockdown of TRA2A and TRA2B from publicly available data^33^. Exons are all coding exons from the longest coding transcript per gene with a GA-multivalent region, categorised based on whether the exon overlaps a GA-multivalent region. e, The proportion of exons spliced in (PSI) in the control samples, using the same data and categories as in panel d. f, Using all available human tissues from VastDB, the percentage of exons in each tissue that are constitutive (PSI > .95, groups as defined in panel d). g, Western blot of the knockdown efficiency of TRA2A and TRA2B siRNA after 36 h. For gel source data, see Supplementary Fig. 1a. h, Quantification of TRA2B knockdown efficiency from immunofluorescence in cells used for HCR-FISH experiments. i, CLIP crosslinking frequency to each 5-mer and the mean percentile of the GeRM score of crosslinked 5-mers for UPF1. j, as in i, but for TRA2B. Source data

Next, we gathered public iCLIP and eCLIP datasets^32–36^ and identified RBPs that preferentially bind to multivalent regions in the CDS. To identify RBPs with preference for multivalent sequences, we determined whether RBPs preferred to bind to a motif when it was in a highly multivalent context, rather than when the same motif was surrounded by dissimilar sequences. For example, UPF1 shows no bias towards multivalency for its most bound motifs, whereas TRA2B strongly prefers to bind to its most bound motifs in a multivalent context (Extended Data Fig. 8i,j). This identified 34 CLIP datasets in which protein had a preference to bind its favoured motifs in a more multivalent context (Fig. 5a, *y* axis). To determine the preferences for specific types of multivalencies, we then compared the density of crosslink sites within each type of CDS GeRM region to the rest of the CDS of that transcript, which showed that each type of GeRM region shows enriched binding of specific sets of RBPs (Fig. 5a). The GA-rich multivalent regions were bound by a large number of RBPs that have been observed to be enriched in nuclear speckles^32,37,38^ (Fig. 5a, bold), including six SR proteins that preferentially assemble on GA-rich GeRM regions, with the TRA2 SR proteins showing the strongest enrichment on these regions.

For example, TRA2 and several other SR proteins bind strongly and specifically to the GA-rich GeRM region that encodes the charged disordered domain of APP (Fig. 5b). We also performed iCLIP for endogenous TRA2B from mouse embryonic stem cells, which confirmed the strong enrichment around the GA-rich GeRM regions of CDSs (Extended Data Fig. 8a,b), and additionally showed that iCLIP reads were more likely to contain a splice junction when the protein was bound to a GA-rich exon, confirming that TRA2B remains bound to multivalent sites post-splicing (Extended Data Fig. 8c). Thus, analyses of reporter and CLIP experiments provide independent lines of evidence implicating TRA2 proteins as likely effectors of interstasis in nuclear speckles.

TRA2 proteins are known to enhance splicing of GA-rich exons^33^. We therefore analysed public data in which TRA2A and TRA2B were co-depleted, which increases skipping of both alternative and constitutive exons^33^, but found no trend for skipping of the exons with GA-multivalent GeRM regions (Extended Data Fig. 8d,e). Moreover, data from VastDB^39^ demonstrate that the GA-multivalent exons from the mRNAs that are retained upon interstasis are highly constitutive across all available human tissues (Extended Data Fig. 8f). In addition, our randomized reporter analysis did not show any link between GA-rich exons and splicing changes of either the exons or their flanking introns during interstasis (Supplementary Fig. 2f). We conclude that binding of SR proteins to highly multivalent GA-rich exons is rarely associated with the modulation of alternative splicing.

For a protein to be an effector of the interstatic dosage loop, it should be sensitive to the dose of R-MCD proteins. Indeed, TRA2B was diffuse throughout the nucleoplasm in the absence of PPIG_LCD_ expression, with only mild colocalization with the nuclear speckle marker SC35, but became enriched in nuclear speckles 16 h after the induction of PPIG_LCD_ (Fig. 5c,d). The degree of speckle localization correlated with the dose of PPIG_LCD_, similar to retained GA-rich mRNAs. The same dose variations of R-MCD protein induced speckle retention of TRA2B and nuclear GA-rich mRNA to a similar extent, indicating that TRA2B could be an effector that drives the sequestration of GA-rich mRNAs in the nuclear speckle.

To demonstrate the role of TRA2 proteins in modulating selective purinergic mRNA sequestration, we performed short-interfering (siRNA)-mediated depletion of TRA2A and TRA2B in cells expressing PPIG_LCD_ (Extended Data Fig. 8g,h and Supplementary Fig. 1a). Using HCR-FISH of *EIF3A* to examine the degree of GA-rich transcript retention in nuclear speckles, we showed a dose-dependent relationship of the effect of TRA2 proteins on *EIF3A* mRNA localization. Cells were grouped by their PPIG_LCD_ expression intensity to ensure that observed effects were not attributed to changes in PPIG_LCD_ expression driven by TRA2 depletion itself. At lower levels of PPIG_LCD_ expression that are sufficient to promote the retention of *EIF3A* mRNA in nuclear speckles in control conditions, double *TRA2A/B* knockdown significantly decreased speckle retention of *EIF3A* (Fig. 5e,f). When R-MCD concentrations become very high, the effect of *TRA2A/B* knockdown diminished, indicating that at such concentrations, other RBPs that assemble on GA-rich multivalent regions could also drive speckle mRNA retention (Fig. 5a,b). This suggests that TRA2 proteins are key effectors of interstasis upon modest accumulation of R-MCDs, which more probably represents physiological variation.

**Extended Data Fig. 9:**
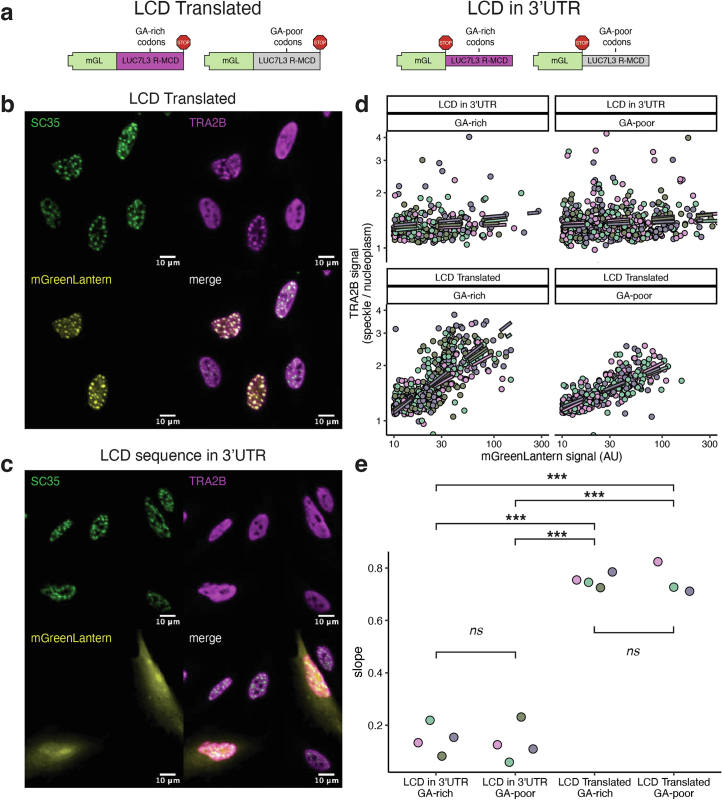
Sequestration of TRA2B in the speckle is driven by R-MCD proteins rather than GA-rich mRNAs. a, A schematic of the mGreenLantern constructs used for the experiments presented in the rest of the panel. b-c, A representative image showing TRA2B and SC35 signal in cells expressing mGreenLantern either with the GA-rich LCD of LUC7L3 as a 3’UTR sequence or as a translated sequence. d, Quantification of the enrichment of TRA2B signal within nuclear speckles versus nucleoplasm per nucleus with respect to mGreenLantern expression for the different constructs shown in panel a. Independent replicates are plotted in different colours and regression lines are plotted in dashed lines. e, The slopes of linear regression models of the relationship between mGreenLantern expression and TRA2B signal within the speckle for the different constructs shown in panel a. Pairwise significance testing are FDR-corrected Welch t-tests, where * = p < 0.05, ** = p < 0.01, *** = p < 0.001. Precise p-values are found in Source Data. Source data

To determine whether the R-MCD proteins or their GA-rich mRNA itself drives the relocalization of TRA2B to the nuclear speckle, we created a construct encoding mGreenLantern fused to the R-MCD of LUC7L3 (Extended Data Fig. 9a). Two versions of each construct were made with opposing codon biases in LUC7L3, to create either GA-rich or GA-poor sequences (Extended Data Fig. 9a). Upon transfection of the constructs, the LUC7L3 R-MCD protein localized to nuclear speckles, and it drove the speckle localization of TRA2B regardless of whether it was expressed from GA-rich or GA-poor sequences (Extended Data Fig. 9b,d,e). We also created a construct in which a stop codon had been inserted upstream of the GA-rich sequence that would otherwise encode the LUC7L3 R-MCD, thus making it part of the 3′ UTR, and this construct had no effect on TRA2B localization (Extended Data Fig. 9c–e). This confirmed that accumulation of R-MCD proteins, rather than their mRNAs, drives TRA2B relocalization, and TRA2B promotes the sequestration of GA-rich mRNA into the speckles.

## CLK kinases modulate interstasis

Localization of SR proteins within nuclear speckles is regulated by their phosphorylation via various kinases, such as the CDC2-like (CLK) family of kinases^40,41^. Previous work has indicated that serine phosphorylation in SR domains disrupts arginine-driven self-association in nuclear speckles^10^. CLK kinase activity is regulated under a diverse array of physiological contexts, including during the cell cycle and in response to small variations in temperature^42,43^. Given the role of TRA2, and probably other SR proteins, as mediators of interstasis, we asked whether the set point of interstasis could be modulated by the activity of CLK kinases.

**Fig. 6:**
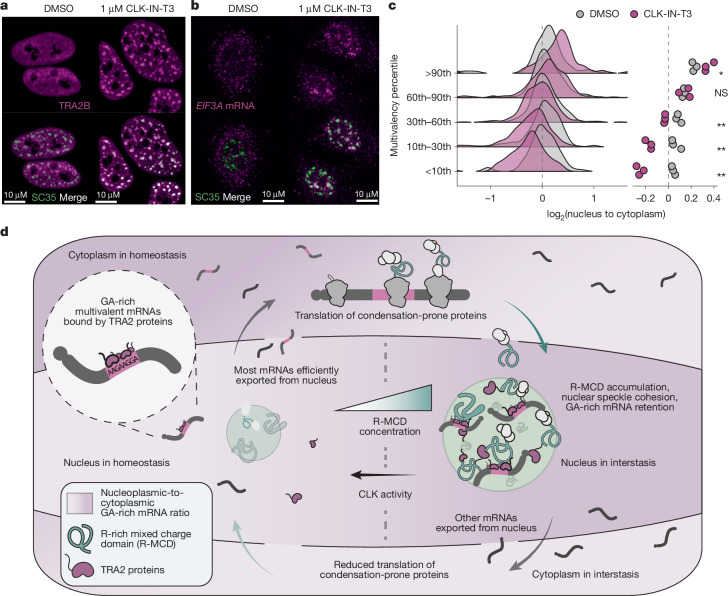
Modulation of interstasis by CLK kinases. **a**, TRA2B and SC35 immunofluorescence in cells treated either with DMSO or 1 µM CLK-IN-T3 for 8 h. **b**, Example image of SON immunofluorescence and *EIF3A* HCR-FISH in cells treated either with DMSO or 1 µM CLK-IN-T3 for 16 h. **c**, The nuclear–cytoplasmic distribution of reporter transcripts binned by their total GeRM scores. Data from cells treated either with DMSO or 1 µM CLK-IN-T3 for 2 h before the induction of the reporter transcript pool for 6 h. The right panel shows the group means per sample. **d**, Schematic detailing the interstasis of proteins with charged low-complexity domains. All statistical comparisons were performed using a Welch *t*-test with FDR correction for multiple testing, where *P < 0.05 and **P < 0.01. Precise *P* values can be found in the Source Data. The black dashed line represents no change between conditions. Source data

**Extended Data Fig. 10:**
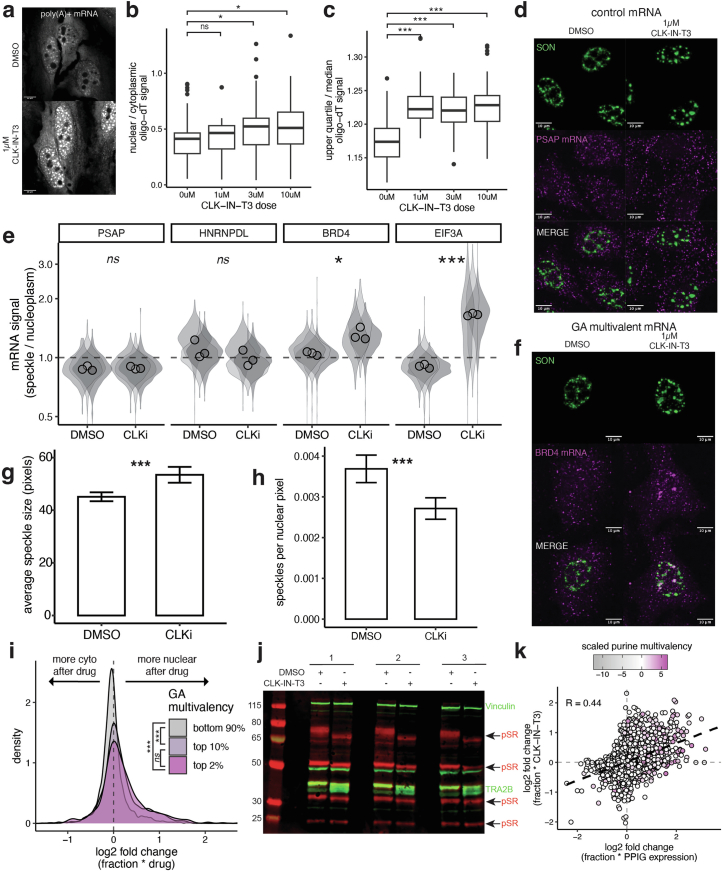
CLK signaling promotes sequestration of GA-multivalent mRNAs in the nuclear speckle. a, Oligo-dT FISH in cells treated either with DMSO or 1 µM CLK-IN-T3 for 8 h. b, The nuclear-cytoplasmic ratio of oligo-dT signal per cell at different doses of CLK-IN-T3 for 8 h. c, The ratio of upper quartile to median oligo-dT signal per nucleus at different doses of CLK-IN-T3 for 8 h. d, Example image of SON immunofluorescence and HCR-FISH of control (PSAP) mRNA in cells treated either with DMSO or 1uM CLK-IN-T3 for 16 h. Scale bars are 10 µm. e, Quantification of the enrichment of HCR-FSH signal within nuclear speckles versus nucleoplasm per nucleus per replicate for control (PSAP, HNRNPDL) or GA-multivalent (BRD4, EIF3A) mRNAs. f, as in d, but with GA-multivalent (BRD4) mRNA. g, The average size of speckles per nucleus per replicate in cells treated with either DMSO or 1 µM CLK-IN-T3 for 16 h. h, The number of speckles normalised by nuclear area per nucleus per replicate in cells treated with either DMSO or 1 µM CLK-IN-T3 for 16 h. i, Per gene interaction effect of subcellular fraction and drug treatment from 3’ end sequencing of nuclear and cytoplasmic fractions of cells treated with DMSO or 1uM CLK-IN-T3 for 8 h. Genes are classified based on the amount of GA-multivalency that they contain in their coding sequences. j, Western blot showing changes in TRA2B phosphorylation (green, lower band) and pan-SR protein phosphorylation (red) following CLK-IN-T3 treatment. For gel source data, see Supplementary Fig. 1b. k, Per-gene log-transformed fold-changes for the interaction terms from PPIG_LCD_ overexpression (12 h doxycycline) or 1 µM CLK-IN-T3 (8 h) where a positive fold change means that the gene was more nuclear after treatment. Points are coloured by their scaled GA-multivalency. Dashed line denotes the linear model fit to the log-transformed fold changes. Pairwise significance testing are FDR-corrected Welch t-tests, where * = p < 0.05, ** = p < 0.01, *** = p < 0.001. Precise p-values are found in Source Data. Source data

We treated HeLa cells for 8 h with 1 µM CLK-IN-T3, a selective inhibitor of CLK1, CLK2 and CLK3 (ref. ^44^), and confirmed that TRA2B and other SR proteins became less heavily phosphorylated (Extended Data Fig. 10j and Supplementary Fig. 1b). We observed that CLK-IN-T3 treatment induced a strong relocalization of TRA2B to nuclear speckles, and a mild enrichment of poly-A^+^ mRNA in the nucleus, especially within speckles (Fig. 6a and Extended Data Fig. 10a–c). Upon 16 h of CLK-IN-T3 treatment, speckles became larger and fewer in number (Extended Data Fig. 10g,h). Thus, CLK kinase inhibition produces a similar phenotype to the overexpression of an R-MCD with regards to mRNA and TRA2B localization and nuclear speckle morphology.

To test the selectivity of speckle mRNA localization, we repeated HCR-FISH after 16 h of CLK-IN-T3 treatment, which significantly increased the localization of the GA-rich *EIF3A* and *BRD4* mRNAs, but not control mRNAs, to nuclear speckles relative to the nucleoplasm (Fig. 6b and Extended Data Fig. 10d–f). To assess the sequence-specific effect on mRNA retention using our reporter system, we treated cells with 1 µM CLK-IN-T3 or DMSO for 2 h, then induced the expression of the reporter transcript pool with doxycycline for 6 h, at which point the expression of the PPIG_LCD_ protein is still negligible, and performed targeted sequencing of the reporter mRNAs. We found that CLK inhibition significantly increased the nuclear retention of the GA-multivalent reporter transcripts (Fig. 6c). To assess the effect on the nuclear–cytoplasmic distribution of endogenous mRNAs, we performed 3′ end sequencing of nuclear and cytoplasmic fractions after 8 h of CLK-IN-T3 treatment, which increased the nuclear retention of mRNAs with GA-rich GeRM regions in CDSs (Extended Data Fig. 10i). The extent of nuclear retention of each transcript was correlated between 8-h CLK inhibition and 12-h PPIG_LCD_ expression (Extended Data Fig. 10k), indicating that both R-MCD abundance and CLK activity can modulate the retention of multivalent mRNAs in nuclear speckles.

## Discussion

We report the discovery of interstasis, a collective feedback loop mediated by a protein–RNA condensate that promotes the homeostasis of a broad range of condensation-prone proteins. Most of these are proteins that interact with RNA, DNA and chromatin, which tend to be enriched in IDRs across all domains of life^45^. Using a novel combinatorially assembled reporter strategy to study mRNA selectivity upon interstasis, we validated the importance of codon biases in the nuclear retention of multivalent mRNAs that encode condensation-prone proteins. Our findings suggest a model in which nuclear mRNA retention is promoted by multivalent assemblies of specific RBPs that localize to nuclear speckles, whereas EJC-dependent packaging counteracts such mRNA capture. We showed that upon increasing the dosage of condensation-prone proteins in speckles, TRA2B relocalizes to speckles, acting as a dosage effector that promotes the capture of multivalent mRNAs (Fig. 6d). By managing the most condensation-prone nuclear proteins that tend to localize into speckles, interstasis probably protects speckles from reaching the toxic levels of cohesion that could non-specifically capture mRNAs^10^. Thus, the properties of a condensate can serve as the long-sought mechanistic link between dosage sensing of IDR-containing proteins and dosage effectors that control their gene expression.

The multivalency of CDS regions encoding IDRs emerges partly due to the structure of the genetic code, that is, the high similarity of codons for biophysically similar amino acids, which tend to cluster within IDRs. Moreover, we found that codon biases further increase such RNA multivalency, especially in the transcriptomes of land vertebrates. All classes of multivalent CDS regions contain strong codon biases that reinforce multivalency, and each class is bound by specific RBPs and encodes IDRs within functionally coherent sets of proteins, indicating a broad potential for their co-regulation. Although autologous interaction between RBPs and their own mRNAs have been linked to affinities between amino acids and their cognate codons^46,47^, broad feedback regulation of a class of related IDRs and their mRNAs, or the role of codon biases in reinforcing such feedback have not been previously observed. Understanding how codon biases might enable feedback regulation between specific sets of proteins and their multivalent mRNAs has implications for various fields, such as interpreting the effect of synonymous mutations on human diseases^48^, and for the design of synonymous gene-recoded therapeutics^49^.

Nuclear retention of several spliced mRNAs has been identified in human tissues^50^, and phosphorylation of SR proteins has been shown to promote speckle mRNA retention; however, the selectivity of such retention remained unclear^51^. We found that the activity of CLK family kinases modulate selective speckle retention of highly multivalent, GA-rich mRNAs. Eight-hour inhibition of CLK kinases phenocopies the same selectivity of mRNA retention as the increased R-MCD expression, as it leads to hypophosphorylation of SR proteins such as TRA2B, which we found to be a key effector of the GA-rich mRNA sequestration into nuclear speckles. CLK signalling is responsive to various physiological states, including cell-cycle progression and small variations in temperature^42,43^, and is investigated as a therapeutic target for many diseases^40^, indicating a broad potential for altered signalling to modify the threshold of interstasis in specific cellular contexts.

The roles in the compartmentalization of biochemical reactions, such as the capacity of speckles to promote the efficiency of splicing, have been the focus of the functional studies of condensates^27^. It has been proposed that an additional function of endogenous condensates could be to buffer cellular noise due to their concentration-dependent assembly^52^, but it remained unclear how heterotypic interactions between diverse sets of proteins and RNAs could contribute to such buffering^53^. We now identify such a function for nuclear speckles, which serve as collective sensors of the combined dosage and modifications of co-condensing proteins. By capturing the mRNAs encoding these proteins, speckles promote the dosage homeostasis of proteins that are expressed from some of the most bidirectionally dosage-sensitive and mutationally constrained genes. It remains to be seen whether nuclear speckles thereby help to protect proteostasis during conditions that affect the relative concentrations of proteins in nucleoplasm, or in diseases with aberrant protein condensates, thereby complementing known pathways for protein quality control that primarily sense cytoplasmic disturbances^54^. Many age-related diseases, especially in neurodegeneration, involve aberrant protein–RNA condensates that can eventually promote aggregation of IDR-containing proteins^4,55,56^, and are often caused by low-complexity repeat expansions that can involve speckle recruitment of the repeat RNA^57,58^. Understanding the mechanisms that manage the homeostasis of proteins in condensates could open new therapeutic opportunities for these diseases^55^. In summary, we have demonstrated a new principle of a ‘collective autoregulatory protein–RNA circuit’ controlled by the mesoscale properties of a condensate.

## Methods

### GeRM algorithm

GeRM is calculated from a string of consecutive overlapping nucleotide sequences of length *k* (*k*-mers). From the set of *n* consecutive *k*-mers *A*, the GeRM score *g* of the central *k*-mer *A*_0_ is calculated as follows:

$$g=\sum _{i\in B}{e}^{-\lambda d({A}_{0},{A}_{i})}\cdot \frac{|w-i|}{w}$$

where *B* = *−w*,…,*−k*,*k*,…,*w*

where $w=\frac{n+1}{2}$, the function *d* represents the Hamming distance, and *λ* represents a scaling constant.

In non-mathematical terms, the GeRM score is calculated by comparing a *k*-mer to all the other *k*-mers that surround it in a fixed window. For each of the surrounding *k*-mers, the sequence similarity to the central *k*-mer is calculated from the negative exponent of the Hamming distance, such that *k*-mers with identical sequences have a high score and those with unrelated sequences have a low score. The constant *λ* determines how quickly this similarity score decays as sequences become more dissimilar to the central *k*-mer. This sequence similarity score is multiplied by a distance score, which decays linearly from 1 to 0 with distance from the central *k*-mer. *k*-mers that overlap with the central *k*-mer are ignored. For *k*-mers at the edges of transcripts, where the window exceeds the end of the transcript, all positions that fall outside of the transcript are given a score of 0. The sum of all the distance-weighted sequence similarities is summed to give the GeRM score.

In this Article, we used a *k*-mer length (*k*) of 5, a window size (*n*) of 123 and a scaling factor (*λ*) of 1. We normalized the GeRM scores such that the minimum value in the transcriptome was 0 and the median was 1. Smoothing of GeRM scores was performed by taking the mean of values in a sliding window of size 123.

For the calculation of codon-shuffled multivalency, all codons for a given amino acid in each transcript were swapped such that the number of instances of each codon was preserved for each transcript, but their order was randomized. All GeRM scores for each transcript were calculated, and the mean GeRM score per position across ten shuffles was calculated.

The GeRM algorithm is available as an R package from GitHub (https://github.com/ulelab/germ).

### Identification and classification of GeRM regions

For each protein-coding gene in GENCODE 29 or GENCODE M22, the transcript with the longest CDS was selected and ties were broken by the longest total transcript length. Only spliced transcripts were used for analysis. CDS GeRM regions were defined by taking all positions where the smoothed GeRM scores exceeded the 98th percentile of smoothed GeRM scores within the CDS, and GeRM regions were adjusted to contain all *k*-mers that fell within the smoothing window. GeRM regions with at least 41 nt of overlap were merged. GeRM regions for which at least one-third of the region fell within an untranslated region were excluded.

For each GeRM region, the relative proportion of the total multivalency of the region that was accounted for by each possible *k*-mer was calculated. For clustering, UMAP was used to reduce the dimensionality of the data to 4 dimensions, using 50 neighbours and a minimum distance of 0.001. The reduced data were clustered first using OPTICS with the minimum points defined by 1% of the total number of GeRM regions. The final clustering was performed with DBSCAN, yielding eight clusters. The reachability threshold for DBSCAN was defined by determining how the proportion of points below a given threshold changed as the reachability threshold decreased (from the 99th percentile to 0), then taking the point at which the first derivative was 40% of the maximum (the knee of the plot). GeRM regions falling outside of a cluster were excluded from further analysis. For presentation purposes, UMAP was used to reduce the original data to have two dimensions, with other parameters kept the same.

Codon shuffling was performed by shuffling the codons either within the entire transcriptome or within each transcript. Shuffling was performed five times, and GeRM scores were calculated from the shuffled sequences. The mean GeRM score per *k*-mer across the five shuffles was used for further analysis.

To generalize the scoring of identified multivalent types (that is, the GA-multivalency score and transcript multivalency), we summarized each GeRM cluster with the *k*-mers that contributed most to multivalency of regions in that cluster. The proportion of total multivalency accounted for by each 5-mer was calculated, and 5-mers were sorted in descending order based on this proportion. The cumulative sum was calculated, and all 5-mers that accounted for the first 50% of the multivalency in each GeRM cluster were selected as representative 5-mers for this multivalency type.

### Low complexity and disorder

To define the complexity of amino acid sequences, information entropy was calculated using the R package HDMD in a sliding window of 41 amino acids along the translated CDSs from the set of transcripts used for GeRM scoring. LCDs were defined as regions with entropy in the bottom 2% of all entropy scores, with all amino acids in the sliding window being considered part of the LCD. LCDs that overlap were merged. AlphaFold^59^ predicted local distance difference test (pLDDT) scores were obtained for the same set of proteins where available from UniProt.

### GeRM codon ratios

To determine whether a given position in a sequence supported the local multivalency of that sequence, we defined a GeRM codon ratio. First, ‘mutable positions’ were defined as any position that could be synonymously mutated. Primarily, these are the final position of the codon, but the first positions of some leucine and arginine codons can also be synonymously mutated. For each mutable position, sequences were produced in which every possible synonymous mutation was made. The GeRM scores of all 5-mers that overlapped with the mutable position were calculated, and the maximum of these GeRM scores was taken for each possible mutation or the native sequence. Then, the maximum GeRM score associated with the 5-mers overlapping the native sequence was divided by the mean of the maximum GeRM scores associated with all possible mutations to create a ratio. When this ratio is positive, the native nucleotide at the mutable position is associated with higher GeRM than the other possible mutations that could exist at that position on average.

### Conservation of GeRM

PhyloP conservation across 100 vertebrates and 470 mammals in hg38 coordinates were downloaded from the UCSC Golden Path. These values were converted into transcriptomic coordinates based on the single transcripts per genes that were used for GeRM calculation. Mutable positions were binned based on their GeRM codon ratio with the binning calculations being applied to each codon separately, such that each bin contained an equal proportion of each codon.

For raw conservation analysis, the average PhyloP conservation values of mutable positions in each GeRm codon ratio bin were used. For analysis of normalized conservation values, the PhyloP of mutable positions were normalized for each codon by subtracting the median conservation of that mutable position in that codon by the median conservation across all mutable positions of the same type. For example, the conservation value associated with one instance of the A in the codon CGA would be normalized by subtracting the median conservation of all As in CGA codons. Each normalized position was then further normalized by subtracting the PhyloP value of the middle position of the codon (in the previous example, the G in that specific instance of the CGA codon). Subtraction was used for normalization because PhyloP scores operate on a logarithmic scale.

### Arginine codon usage

For analysis of arginine codon usage in R-LCDs, all human LCDs were filtered for those that obtain at least 20% arginine residues. The proportion of each arginine codon in the respective coding regions was calculated. Pairwise correlations between arginine codon usages using Spearman’s rank correlation. To define CG-rich and GA-rich codon groups, principal component analysis was performed based on the arginine codon usage for all R-LCDs, and the top and bottom third of LCDs based on the first principal component were defined as CG-rich and GA-rich R-LCDs, respectively.

For the definition of R-LCDs in different species, all CDSs for each species were downloaded from Ensembl and a single transcript per gene was identified according to the previously described approach. LCDs encoded by these sequences were defined as previously described, using the complexity threshold defined for the human proteome for each species. A full list of the species and annotation versions can be found in Supplementary Table 1.

### Gene Ontology analysis

Gene Ontology analyses were conducted using the topGO R package and using the weight01 algorithm to account for the topology of the Gene Ontology graph.

### Cell culture

HeLa cells were obtained from Cell Science at The Francis Crick Institute and cultured in DMEM + GlutaMAX (Thermo Scientific) with 10% FBS and split every 3–4 days using TrypLE Express (Thermo Scientific). Low-passage, wild-type mouse embryonic stem cells (mESCs; 129S8/B6 background) were obtained from the Genetic Modification Service at The Francis Crick Institute and cultured feeder free on Nunc cell culture dishes (Thermo Scientific) coated with 0.1% gelatin (ES-006-B, Millipore). mESCs were maintained in 2i media^60^, fed every day and split every 2–3 days using Accutase (A6964, Sigma). All cell lines were sourced from, authenticated by and mycoplasma tested at The Francis Crick Institute.

All cell transfections were performed using Lipofectamine 3000 (Thermo Scientific) according to manufacturer’s instructions. Doxycycline induction was performed by changing cell culture media to fresh, pre-warmed media containing 100 ng ml^−1^ doxycycline. CLK-IN-T3 (SML2649, Sigma) was dissolved to 5 mM in DMSO. In experiments using CLK-IN-T3 treatments, control cells were treated with the equivalent volume of DMSO.

### Plasmid construction

Double-stranded DNA fragments were ordered as gBlocks or eBlocks from IDT. Primers were ordered from Sigma-Aldrich. All PCRs utilized Q5 High-Fidelity Master Mix (NEB) or Phusion High-Fidelity Master Mix (NEB). Backbone plasmids were linearized via PCR followed by treatment with DpnI (NEB) before assembly with fragments using NEBuilder HiFi DNA Assembly (NEB) according to the manufacturer’s protocol. Phosphorylations and ligations were performed using T4 PNK kinase (NEB) and T4 DNA ligase (NEB). Assembly reactions were purified using magnetic SPRI beads (Mag-bind TotalPure NGS; Omega-Bio-tek) before DNA transformation into 5α competent *Escherichia coli* (NEB). All sequences were confirmed via Sanger sequencing (Source Bioscience). pTwist-AMP high-copy plasmid was purchased from Twist Bioscience and was used as a vector for all overexpression via transient transfection. Sequences of all LUC7L3 constructs can be found in Supplementary Table 5.

For the three additional R-MCD plasmids, the following regions of LUC7L3_221–415_, PRPF38B_251–534_ and SRSF11_171–389_ were fused to mGreenLantern and assembled into pTwist-CMV plasmid backbones.

### Subcellular fractionation

Nuclear–cytoplasmic fractionation was carried out by trypsinizing whole cells and washing twice with PBS. Cells were then pelleted and resuspended in 200 µl cytoplasmic lysis buffer: 50 mM HEPES pH 7.5, 2 mM MgCl_2_, 50 mM 2-mercaptoethanol, 0.025% NP-40, 0.05% saponin and 1X cOmplete EDTA-free protease inhibitors (Sigma). Cells were rotated at 4 °C for 10 min, pelleted and the supernatant was taken as the cytoplasmic fraction. The nuclear pellet was washed in 1 ml cytoplasmic lysis buffer for 3 min, then pelleted again. The supernatant was removed and the nuclei were lysed in 200 µl iCLIP lysis buffer^61^ to obtain nuclear fractions. All centrifugations were performed at 4 °C and at 300*g* for 3 min. All buffers were kept ice cold. For each fraction, 20 µl was used for western blotting to confirm the efficiency of the fractionation, which was stable across all samples (Extended Data Fig. 4a, Supplementary Fig. 3). In all western blots, vinculin was used as a loading control (1:2,000; 700062, Thermo Scientific). Anti-phosphoepitope SR proteins (1:1,000; MABE50, Sigma) were used to detect SR phosphorylation changes.

### RNA extraction

RNA extractions were performed from cell pellets using the Maxwell RSC simplyRNA kit (Promega) using a Maxwell RSC Instrument (Promega).

### 3′ End sequencing

For each sample, 250 ng µl^−1^ of RNA was fragmented in 10 mM Tris-HCl pH 7.5 and 10 mM MgCl_2_ buffer for 5 min at 95 °C. Of fragmented RNA, 2 µl was mixed with 2 µl water, 0.5 µl of 10 mM dNTPs and 0.5 µl of 5 µM of oligo-dT reverse transcription primers containing Illumina P7 sequences, and primers were annealed by heating at 65 °C for 3 min then cooling to 42 °C at 1 °C per second. At this point, reverse transcription was carried out using SuperScript IV (Invitrogen) according to manufacturer’s instructions, with the addition of 0.25 µl of 40 µM of a template-switching oligo containing Illumina P5 sequences and unique molecular identifiers (UMIs). The reaction was incubated for 1 h at 42 °C. Following alkaline hydrolysis of RNA, the product was purified using magnetic SPRI beads and amplified using 0.5 µM i5/i7 Illumina indexed primers in Q5 High-Fidelity Master Mix (NEB).

### Analysis of RNA-seq data

All sequencing data were mapped to the GRCh38.p12 genome using the GENCODE v29 basic genome annotation. All 3′ end sequencing data were trimmed using cutadapt (v4.4)^62^ to remove poly-A sequences and Illumina adapters. All data were processed using the nf-core RNA-seq Nextflow pipeline (v3.12.0)^63^, and in the case of the 3′ end sequencing data, the additional option --noLengthCorrection was provided to Salmon to prevent length correction for gene expression.

Differential expression analysis was conducted using DESeq2 in R^64^, with effect size shrinkage using the apeglm package^65^. Changes in the nuclear:cytoplasmic ratio between two conditions were assessed using the interaction term of the models (fraction × time). Splicing analysis was conducted using rMATS (v4.1.2)^66^ using the skipped exons calculated using the JCEC quantifications.

### Transcriptome-wide GA-multivalency scoring

To classify genes based on their degree of GA multivalency, a GA-multivalency score was calculated for each gene based on the CDS of its primary transcript, defined by the longest annotated CDS, with ties settled by total transcript length. First, for each of the three previously identified GA-rich GeRM clusters (A-rich and G-rich AG, AG + C), representative *k*-mers were derived as described in the section ‘Identification and classification of GeRM regions’. Then, raw multivalency scores of 5-mers were scaled to all other instances of the same 5-mer, such that their mean score was 0 and their standard deviation was 1. For each transcript, the sum of all these scaled GeRM scores was summed across all representative 5-mers. On the basis of this metric, transcripts that have many instances of GA-rich 5-mers specifically in multivalent contexts are rewarded. All processed data from 3′ end sequencing experiments can be found in Supplementary Table 4.

### pSILAC

Cells were grown for 48 h in lysine-free and arginine-free DMEM (Thermo Scientific), supplemented with 10% dialysed FBS (Thermo Scientific), 600 mg l^−1^ proline and 100 mg l^−1^ arginine and lysine (‘light’ media). Cells were induced with 500 ng ml^−1^ doxycycline for 12 h before switching to either medium arginine ^13^C_6_ (88210, Thermo Scientific) and lysine 4,4,5,5-D4 (DLM-2640, Cambridge Isotope Laboratories; ‘medium’ media) or heavy arginine ^13^C_6_^15^N_4_ (89990, Thermo Scientific) and lysine ^13^C_6_^15^N_2_ (88209, Thermo Scientific; ‘heavy’ media) for 8 h. Media were refreshed every 2 h, with doxycycline treatment maintained throughout.

### Mass spectrometry

Cells were washed with PBS and lysed in 4% SDS, 100 mM Tris-HCl pH 7.5 buffer supplemented with protease inhibitors (Sigma). Samples were next sonicated, and their protein concentrations quantified via DC protein assay (Bio-Rad).

Lysates were reduced by the addition of dithiothreitol to a final concentration of 5 mM, vortexed briefly and incubated at 56 °C for 30 min. Samples were cooled to room temperature and alkylated with iodoacetamide to a final concentration of 20 mM. Following brief vortexing and incubation in the dark at room temperature for 20 min, residual iodoacetamide was quenched with 5 mM dithiothreitol for 10 min. Trypsin was added at a 1:100 enzyme:substrate ratio. Samples were vortexed, centrifuged for 1 min at 14,000 rpm and incubated overnight at 37 °C shaking at 750 rpm.

Peptide samples (1 µg on column) were analysed by liquid chromatography with tandem mass spectrometry (LC–MS/MS). Chromatographic separation was performed using the U3000 UHPLC NanoLC system (Thermo Scientific) and peptides were resolved by reversed phase chromatography on a 75-µm C18 Pepmap column (50 cm length) via a three-step linear gradient comprising 80% acetonitrile in 0.1% formic acid. The gradient was delivered to elute peptides at a flow rate of 250 nl min^−1^ over 120 min, initially at 4% solvent at 0–10 min, then increasing to 30% solvent (10–75 min) and 40% solvent at 75–80 min. This was followed by a wash step with 99% solvent (85–90 min) and a final equilibration step with 4% solvent (90–120 min).

Peptides were ionized by electrospray via an Orbitrap Fusion Lumos mass spectrometer (Thermo Scientific) operating under Xcalibur (v4.3). Data were acquired in data-dependent acquisition mode using an Orbitrap-Ion Trap method with a 3-s cycle time between a full MS scan and MS/MS fragmentation by collision-induced dissociation. Orbitrap spectra were collected at 120,000 resolution over an *m*/*z* range of 350–1,600, with an automatic gain control of 4.0 × 10^5^ (100%) and maximum injection time of 35 ms. Monoisotropic precursor selection was enabled for charge states +2 to +5 with an intensity threshold of 5 × 10^3^–1 × 10^20^ and dynamic exclusion of 35 s ± 10 ppm. MS2 precursor ions were isolated in the quadrupole with a mass width filter of 1.6 *m*/*z*. Ion trap fragmentation spectra were collected with an automatic gain control target setting of 1.0 × 10^4^ (100%) with a maximum injection time of 35 ms with collision-induced dissociation collision energy set at 35%.

Raw mass spectrometry data were processed into peak list files using Proteome Discoverer (v3.1; Thermo Scientific) and searched with the Sequest algorithm^67^ against the Uniprot Human isoform database (188,476 entries; September 2024). This was concatenated with the mScarlet fluorescent protein sequence. Database searching was performed at a stringency of 1% FDR including a decoy search with precursor ion intensity quantification. Post-translational modifications to amino acid residues included carbamidomethylation (C) and variable oxidation (M), SILAC ^13^C_6_ (R) and SILAC ^2^H_4_2 (K).

The proportion of SILAC-labelled peptides for each protein was compared between doxycycline-treated and untreated samples to determine the change in translation or protein turnover. The mean log_2_ fold change for all proteins belonging to different multivalency classes in each replicate was calculated and this per-replicate mean was used for pairwise significance testing. The proportion of the total proteome or total R-MCD abundance attributed to mScarlet–PPIG_LCD_ overexpression was calculated using the peptides assigned to mScarlet, and R-MCDs were classified as previously defined R-LCDs (those containing at least 20% arginine), with the additional constraint that the net charge must be positive and the total fraction of charged residues in the LCD must be greater than 40%.

### Immunofluorescence

Immunofluorescence was performed by fixing cells in 4% paraformaldehyde and 0.1% glyoxal at room temperature for 10 min. Samples were permeabilized by incubating with PBS with 0.5% Triton-X for 5 min at room temperature. Samples were blocked using PBS with 3% BSA and 0.1% Tween (blocking solution). Primary antibody incubations were carried out in blocking solution for 1 h at room temperature or overnight at 4 °C. Samples were washed three times with PBS with 0.1% Tween for 5 min, and secondary antibody incubations were carried out in blocking solution for 1 h at room temperature in the dark. Samples were washed twice, then incubated with 200 ng ml^−1^ DAPI for 15 min in the dark at room temperature. DAPI was washed out and samples were placed in glycerol mounting medium (90% glycerol, 20 mM Tris pH 8 and PBS).

The following antibodies were used at the following concentrations: rabbit anti-TRA2B (1:1,000; ab31353, abcam), rabbit anti-SON (1:1,000; HPA023535, Sigma) and mouse anti-SC35 (1:500; S4045, Sigma). Secondary antibodies to mouse and rabbit, conjugated to Alexa Fluor 488 or Alexa Fluor 647 were used at 1:500 dilution (Abcam). This included goat pAb anti-rabbit IgG Alexa Fluor 647 (ab150079), goat pAb anti-rabbit IgG Alexa Fluor 488 (ab150077), goat pAb anti-mouse IgG Alexa Fluor 488 (ab150113) and goat pAb anti-mouse IgG Alexa Fluor 647 (ab150115).

### HCR-FISH

For each mRNA, HCR 12 probe pairs were designed with the B4 amplifier sequences using the HCR 3.0 Probe Maker^68^. HCR 3.0 was performed on cells in µ-Slide eight-well glass bottom slides (80827, Ibidi) as described in the original protocol^69^, with the following modifications. First, fixation and permeabilization was performed as described in the immunofluorescence section. After pre-hybridization, primary probe hybridization was performed with a probe concentration of 10 nM for 3 h. Overnight HCR amplification was performed in a volume of 125 µl with half the concentration of HCR hairpins (Molecular Instruments). Following HCR amplification, samples were washed according to the original protocol, and then immunofluorescence was performed as previously described, but with all buffers containing 2XSSC in place of PBS.

In all cases of R-MCD overexpression transfections, HCR-FISH was performed on HeLa cells 24 h post-transfection with 50 ng of mGreenLantern-fused R-MCD plasmid, in combination with 200 ng of pUC19 filler DNA.

### oligo-d(T) FISH

Samples for oligo-d(T) FISH were fixed as described for immunofluorescence. Samples were incubated with HCR 3.0 hybridization buffer for 30 min at 37 °C, then in hybridization buffer containing 1 µg ml^−1^ oligo-dT(25)-Cy5 for 2 h. Samples were washed twice in 5XSSC with 0.1% Tween, stained with 200 ng ml^−1^ DAPI in the same buffer and washed once more before glycerol mounting medium was added.

### Microscopy and image analysis

Microscope images were acquired using an Olympus IX3 Series (IX83) inverted microscope, equipped with a Yokogawa W1 spinning disk and a Hamamatsu Orca Fusion CMOS camera (pixel size of 6.5 μm, 2,304 × 2304, 5.3 megapixels).

All images were *z*-projected using a maximum projection. Segmentation of nuclei and cytoplasms was performed using Cellpose (v2.0)^70^ using the cyto2 model. Segmentation of nuclear speckles was performed using CellProfiler using the Otsu thresholding algorithm, and nucleoplasms were considered all non-speckle regions of the nucleus. The mean signal intensity for HCR-FISH or immunofluorescence within speckles was compared with the mean intensity in the nucleoplasm for each cell to create a speckle enrichment ratio. Mean intensities in the nucleoplasm and nuclear speckle were calculated per cell, but statistical comparisons between groups counted each replicate as a single observation, taking the mean values across all cells in the replicate.

### Features predicting nuclear retention of RNA

Using predictive modelling, we aimed to evaluate the contribution of CDS length, EJC density and different types of multivalent features on nuclear retention of endogenous transcripts in interstasis. To disentangle the contributions of the different multivalent features, we first cleaned the data to reduce collinearities. Representative *k*-mers were assigned for each of the eight GeRM clusters as described in the section ‘Identification and classification of GeRM regions’. Subsequently, *k*-mers belonging to multiple clusters were retained only in the cluster to which they contributed the highest proportion of multivalency, ensuring no *k*-mers overlapped between clusters.

Next, the raw *k*-mer multivalencies for all *k*-mers belonging to a cluster were summed across CDSs of representative transcripts, defined as the transcript with the longest CDS per gene, and normalized by CDS length. These length-normalized multivalency sums were then standard scaled across the transcriptome so that the mean transcriptome-wide multivalency of each GeRM cluster was zero. We assessed the correlations between the multivalencies of the eight GeRM clusters and found a high correlation among the C-rich, CUG-repeat, G-rich, G/C and GA + C multivalent classes. Given this high correlation and the similarity of their *k*-mers, we combined these clusters into a single GC-rich cluster for training. We repeated the scoring with the combined *k*-mers from all these clusters. This approach efficiently minimized collinearities between features, except for a moderate anti-correlation (Spearman’s *r* = −0.75) between the GC-rich and A-rich clusters. Features used for training are summarized in Supplementary Table 8.

Then, we trained a classification model to differentiate between mRNAs retained in the nucleus after PPIG_LCD_ induction and control mRNAs that did not show a significant change in their nuclear-to-cytoplasmic ratio under these conditions. mRNAs were classified as retained if they had a *q* < 0.05 and a log_2_ fold change greater than 1 at 12 h post-PPIG_LCD_ induction, resulting in 275 transcripts. Control mRNAs were defined by a *q* ≥ 0.05 and a log_2_ fold change between −0.1 and 0.1, yielding 1,697 transcripts.

Owing to significant imbalance between the positive (nuclear retained) and the negative (control) class, we used a BalancedRandomForestClassifier from imbalanced-learn module (v0.12.3)^71^, leveraging sampling of the majority class to balance the classes in the process of training (sampling_strategy = ‘not minority’). The classifier was configured with 100 trees (n_estimators = 100) and used bootstrap sampling with replacement to enhance generalization. Gini impurity criterion was used for node splitting and the minimum number of samples required at a leaf node was set to 15. For evaluation of the classifier’s performance, we used fourfold stratified cross-validation. This method splits the data into fourfolds, with each fold serving as a test set once while the remaining threefolds serve as the training set. Stratified *k*-fold cross-validation ensures that each of the folds maintains the same proportion of positive and negative samples as the entire dataset, thereby preventing skewed performance metrics. In this case, the model was trained four times, with each training set comprising 75% of the data and each test set comprising 25%, ensuring balanced class representation in both training and testing. Visualized AUROC metrics were averaged across folds to obtain a robust estimate of the classifier’s performance.

To identify the optimal set of input features and assess their effect on predictive performance, we used a stepwise feature-selection approach. Initially, we trained models with individual features and progressively expanded the feature set, by selecting the best-performing single feature or feature combination and adding the remaining features on top of it. This process was repeated until adding more features no longer enhanced the performance of the model. All models were evaluated using fourfold cross-validation, as described.

### Reporter library design

The mScarlet–PPIG_LCD_ library was designed by concatenating the CDSs for mScarlet and PPIG_402–684_. The concatenated sequence was then iteratively synonymously mutated such that the GC content of the sequence was maintained within 2% of the original sequence, choosing codons with fewer As and Gs until the number of AG-only 5-mers reached a minimum. Untranslated regions were added to this sequence. Seven equally spaced sequences of 28 nt were chosen along the sequence to be used as overlap sequences for future Gibson assembly. These overlap sequences were protected from future in silico mutagenesis.

Twenty-six constitutively spliced introns ranging from 83 nt to 384 nt in length were selected from housekeeping genes. We generated 25 different combinations by randomly choosing 8 of these introns, then performed the following iterative optimization algorithm to each of these 25 combinations to generate sequences with optimized predicted splicing.

First, introns were inserted in the reporter CDS in positions that were exactly between the previously defined overlap sequences or the start and end codons. The probability of donor and acceptor site usage was predicted using SpliceAI, and all probabilities were summed. Next, 100 coding sequences with 5 random synonymous mutations were generated, and a sequence was selected that best maintained GC content and kept GA 5-mer content low. After this, an intronic sequence was selected with a 80% chance to be mutated at 5% of its positions and a 50% chance to be shifted by up to 20 nt. The probability of donor and acceptor site usage in the new sequence was predicted with SpliceAI, and the sum of the probabilities was compared with the previous sequence. If the total probability of splice site usage was increased in the mutated sequence, then the mutated sequence was put forwards for another round of mutation, until 2,000 iterations were completed.

To create a GA-rich version of the reporter CDS, the iterative process was repeated, but with synonymous mutations that maintained GC content but maximized GA-rich 5-mer content. The sequence and position of the introns were kept constant for this process.

To determine which of the 25 possible combinations of 8 introns resulted in the best-predicted splicing in both GA-poor and GA-rich reporter sequences, we selected the intron set with the highest summed probability of predicted donor and acceptor site usage. This resulted in GA-poor and GA-rich codon-biased reporter sequences in which both splice sites for each of the eight introns was predicted to have at least a 98.5% probability of being spliced.

For both the GA-rich and the GA-poor sequence, eight sequence segments were generated that spanned from each overlap sequence to the next (or to the untranslated region) and that either contained an intronic sequence or not. Therefore, each of the eight segments of the final reporter gene varied in two possible ways: GA richness and the presence or absence of an intron, and each of these variable segments shared a fixed overlap sequence with the neighbouring segment.

### Reporter library generation

All 32 possible segment variations were ordered as double-stranded DNA fragments from IDT. In addition to the overlap sequences that would be part of the final reporter transcript, additional overlap sequences were added for intermediate assembly steps. Specifically, an overlap sequence was added to the 3′ end of the first segment and to the 5′ of the last segment, and another overlap sequence was added to the 3′ ends of the first and fifth segments and the 5′ ends of the fourth and last segment. Then, two pools containing all variations of the first four segments or all variations of the last four segments were made, such that intron-containing variants had twice the molarity of intronless variants at each position, but all positions had the same overall molarity. A Gibson assembly reaction was carried out on each pool, and due to the additional overlaps on the first, fourth, fifth and final segments, circular pieces of double-stranded DNA were formed, each containing one of the four variations at each of the four positions used for that pool.

These circular pieces of DNA were then linearized and amplified by PCR, and the linearized fragments deriving from the first and second half of the reporter gene were again Gibson assembled together. Again, because of the additional overlap sequences attached to the first and last segments, the resulting assemblies were circular. Each assembly contained a 5′ UTR, a CDS encoding mScarlet–PPIG_LCD_ but with a variable amount of GA-rich 5-mers and a variable number of introns, and a 3′ UTR. These assemblies were then linearized and amplified by PCR and Gibson assembled into a plasmid backbone (pTwist-CMV) containing a CMV promoter and a barcoded 3′ UTR sequence with a poly-adenylation site. Therefore, the plasmids in the pool contained different versions of the reporter gene, each with a unique barcode in their 3′ UTR.

After each reaction, the product was purified using Mag-Bind TotalPure beads. All PCRs were performed using Q5 High-Fidelity Master Mix (NEB). All Gibson assemblies were performed using NEBuilder HiFi DNA Assembly (NEB).

To generate a doxycycline-inducible PiggyBac integration vector containing the reporter library (ePB-ExportReporter), reporter genes and their barcoded UTRs were amplified out of the original reporter plasmid library with a low number of PCR cycles, before being inserted into an all-in-one PiggyBac plasmid (ePB-Bsd-TT-NIL) containing a doxycycline-inducible promoter as well as a constitutive promoter driving the expression of the rtTA protein and blasticidin resistance gene. All designed fragments, as well as example reporter sequences and plasmids, are provided in Supplementary Table 2.

### Long-read sequencing

For sequencing of the plasmid pool, the reporter genes were amplified from the plasmid DNA with a minimal number of PCR cycles. For sequencing of the reporter RNA, one well of a six-well plate of HeLa cells were transfected with 500 ng of the reporter plasmid pool and 2,000 ng of a filler pUC19 plasmid for 16 h. RNA was extracted and 500 ng of total RNA was used as input for a SuperScript IV reverse transcription (Invitrogen) reaction using an oligo-dT reverse transcription following the manufacturer’s instructions. The reaction was purified with magnetic beads, then amplified according to the same approach as for the plasmid DNA. The specificity of the amplicon was confirmed with an agarose gel. In both cases, the PCR product was purified with magnetic beads (Mag-bind TotalPure NGS, Omega-Bio-tek) and Nanopore adapters were added using the Nanopore Ligation Sequencing Kit (Oxford Nanopore), according to the manufacturer’s instructions. The libraries were sequenced separately using MinION R9.4.1 Flow Cells (Oxford Nanopore).

Raw Nanopore data were basecalled with Guppy (v6.0.1). The basecalled reads were mapped to a genome of possible reporter transcripts using minimap2 (ref. ^72^), and barcodes were extracted by matching the upstream and downstream sequences using the R package stringdist^73^.

### Targeted sequencing and its analysis

Targeted sequencing libraries were prepared from 500 ng of RNA input per sample. A targeted reverse transcription was performed with SuperScript IV (Invitrogen) using a reverse transcription primer that was specific to the reporter 3′ UTR and contained a 4-nt experimental barcode, a 10-nt UMI and an Illumina p7 adapter sequence. The reaction was performed according to manufacturer’s instructions. Samples were multiplexed in groups of six at this point, and the RNA was removed by alkaline hydrolysis for 15 min, the pH was neutralized with hydrochloric acid, and the cDNA was purified with magnetic beads. A nested PCR was then performed in which ten cycles with one set of primers was performed. Of the reaction, 5% was spiked into the next six-cycle reaction, in which an Illumina p5 adapter sequence was added. The PCR was purified with magnetic beads (Mag-bind TotalPure NGS, Omega-Bio-tek), and Illumina i5 and i7 indices were added by a final PCR. Libraries were sequenced on a NovaSeq 6000 by the Advanced Sequencing Facility at The Francis Crick Institute. All primer sequences can be found in Supplementary Table 2.

Targeted sequencing libraries were analysed in R. Libraries were demultiplexed using the barcodes introduced by the reverse transcription primer, and the plasmid barcode was extracted by matching the flanking constant regions using fuzzy string matching using the R package stringdist. Reads containing the same barcode were deduplicated by their UMI sequences. Barcodes were counted and related to their reporter gene characteristics based on the long-read sequencing data. Barcode counts were normalized to the total number of assigned barcodes for each sample. Barcodes with low numbers of counts were excluded from further analysis. Nuclear-to-cytoplasmic ratios for each barcode were calculated for each replicate. All processed data for each experiment can be found in Supplementary Table 3.

### Cell line generation

We cloned the reporter pool into a doxycycline-inducible PiggyBac vector and generated the PiggyBac cell lines by co-transfecting HeLa cells with equal amounts of the ePB-ExportReporter plasmid pool and a plasmid expressing the PiggyBac transposase. A transposase-negative control was also prepared. Cells were selected once with 5 µg ml^−1^ blasticidin for 4 days, then expanded for 1 week, before being selected again with blasticidin until all cells in the transposase-negative control were dead. This pool of ePB-ExportReporter-positive cells was then expanded and stocks were frozen.

### Calculating RBP-binding potential

To understand which RBPs have the potential to bind to GA-multivalent reporters with high affinity, we designed a ‘binding potential’ score, based on RBP-binding motifs obtained with the RNA-Bind-N-Seq method. We downloaded enrichment scores (*R* scores) for 78 RBPs published by Dominguez et al.^30^, and for TRA2B^32^ from the ENCODE portal, yielding motifs for 79 distinct RBPs. Accession codes and relevant concentrations of RNA-Bind-N-Seq experiments are specified in the supplementary material of the original publication (see supplementary table 3 in ref. ^30^). The *R* scores for the RBP concentration that yielded the highest motif enrichment were converted to *z* scores using mean and standard deviation. A tail test was then applied to compute *P* values, and motifs with a *P* < 0.05 were selected for further analysis.

To evaluate the reporter sequences in terms of RBP-binding potential, we divided their CDSs into 5-mers using a rolling window of 5 nt. We then counted the 5-mers that were labelled as significant for a given RBP and multiplied the count for each significant 5-mer by its *z* score. The values obtained were summed into a single score for each RBP (Fig. 5h). The binding potential scores for all evaluated RBPs and reporter sequences can be found in Supplementary Table 9.

### Multivalency preference in public CLIP data

All public CLIP data were processed as previously described^74^, with replicates merged. All crosslinks falling within CDS per sample were counted and the proportion of CDSs to total crosslinks was calculated. Samples in the bottom third were excluded. Remaining samples in the bottom third of raw CDS crosslink counts were also excluded, to remove low-quality datasets. To determine whether a protein tends to bind to RNA in multivalent contexts, the percentile of the GeRM score was calculated for each possible 5-mer in all CDSs. For each crosslink in each sample, the mean GeRM percentile of each crosslinked 5-mer was calculated. If a given 5-mer is crosslinked preferentially in multivalent contexts (relative to the average multivalency of that 5-mer), then the mean GeRM percentile will be high. The mean 5-mer GeRM percentiles for the top 50 most crosslinked 5-mers was compared with all other 5-mers, producing a ratio. This ratio represents the general bias of a protein to bind its preferred motifs in a multivalent context. In the examples shown in Extended Data Fig. 8i,j, this ratio is the mean *y* axis position for the 50 right-most points on the *x* axis over the mean *y* axis position of all other points. Only samples with a ratio of at least 1.1, implying a preference for multivalent motifs, were kept for further analysis.

Only transcripts in the top half of total crosslinks per nucleotide in the CDS across all samples were used for further analysis, as samples with a low crosslinking density typically did not contain enough crosslinks per GeRM region to produce meaningful results. For each GeRM region, the ratio of the number of crosslinks falling within that GeRM region compared with the rest of the CDS for that transcript was calculated. The mean of this ratio within each GeRM cluster was calculated for each sample. Samples were then hierarchically clustered and plotted as a heatmap (Fig. 5a).

### TRA2B iCLIP and its analysis

iCLIP was performed according to the iiCLIP protocol^61^, using crosslinked cell lysate containing 1.5 mg of protein per sample. Two replicates were prepared for each CLIP target. For immunoprecipitation, 5 µg anti-TRA2B (ab31353, abcam) was used. Membrane transfer was performed overnight at 4 °C with reduced voltage (15 V). The entire lane was cut from 40 kDa upwards.

CLIP libraries were trimmed and demultiplexed using Ultraplex^75^ and mapped to a small RNA genome containing all rRNA, small nuclear RNA, tRNA and small nucleolar RNA sequences from GENCODE vM22 using STAR (v2.7.0)^76^. All unmapped reads were then mapped uniquely to the genome using STAR, with the transcriptome-mapping reads output using the flag --quantMode TranscriptomeSAM, and the 5′ end of the read was forced to be aligned using the flag --alignEndsType Extend5pOfRead1. Reads were demultiplexed using UMItools^77^. Crosslink sites were defined as the position immediately upstream of the read.

Metaprofiles around different GeRM regions were created by taking the midpoint of each GeRM region and counting the transcriptome-mapped crosslinks in each position relative to the midpoint. All counts were summed across GeRM regions of the same cluster, then normalized by the number of GeRM regions within the cluster and the number of millions of unique transcriptome-mapping crosslinks in each sample.

### TRA2 siRNA transfection

For siRNA-induced double TRA2 knockdowns, 100 nM of ON-TARGETplus siRNA pools, consisting of four siRNAs targeting both *TRA2A* (LQ-019480-00-0005, Dharmacon) and *TRA2B* (LQ-007278-00-0005, Dharmacon), were transfected using Lipofectamine RNAiMAX (Thermo Scientific) following the manufacturer’s reverse transfection protocol. Transfections with a 100 nM scrambled non-targeting control pool (D-001810-10-05, Dharmacon) were carried out in parallel. Thirty-six hours after transfection, doxycycline was added to the transfection media to induce mScarlet–PPIG_LCD_ expression over a period of 12 h. A total of 48 h post-transfection, cells were either fixed for HCR-FISH as previously described or collected for western blotting to confirm efficient knockdown of *TRA2A* (1:500; H00029896-B01P, Novus Biologicals) and *TRA2B*. This was quantified via ImageStudio 5.x Odyssey Clx (LI-COR).

### Flow cytometry

For each sample, a single well of a six-well plate of ePB-ER HeLa cells was transfected with one of the mGreenLantern LUC7L3-LCD reporter plasmids in which the LUC7L3 sequence was used as a 3′ UTR but was either GA rich or GA poor. Doxycycline was added to the medium at the point of transfection. Four replicates were performed for each plasmid, as well as untransfected and uninduced controls. After 16 h, the cells were dissociated, washed twice with ice-cold PBS and kept on ice while the samples were measured with an LSR Fortessa (BD Biosciences). The intensities of mGreenLantern and mScarlet were measured using a 488-nm blue laser (Coherent Sapphire 100 mW) with a 530/30-nm filter, and a 561-nm yellow green laser (Coherent Sapphire 100 mW) with a 610/20-nm filter, respectively.

Gating of singlets was performed using FlowJo, and subsequent analysis was performed in R. For each plasmid, the mGreenLantern intensities were normalized to the mean intensity values of the uninduced control samples. Cells were binned according to their mScarlet intensity values, and the mean mGreenLantern intensity values for all cells in each bin were calculated per replicate.

### Statistics

All statistics were performed in R (v4.2.0). All tests were two-tailed when possible. All pairwise comparisons were made using a Welch *t*-test unless otherwise stated. When appropriate, *P* values were corrected for multiple testing using the Benjamini–Hochberg procedure.

### Reporting summary

Further information on research design is available in the Nature Portfolio Reporting Summary linked to this article.

## Online content

Any methods, additional references, Nature Portfolio reporting summaries, source data, extended data, supplementary information, acknowledgements, peer review information; details of author contributions and competing interests; and statements of data and code availability are available at 10.1038/s41586-025-09568-w.

## Supplementary information

Supplementary Information Supplementary Figs 1–3.

Reporting Summary

Supplementary Tables Supplementary Tables 1–9 and a Supplementary Table guide.

## Source data

Source Data Figs. 1, 3–6 and Source Data Extended Data Figs. 1, 3, 6–10.

## Data Availability

All sequencing data have been deposited via ArrayExpress. The 3′ end sequencing experiments following PPIG_LCD_ expression and CLK-IN-T3 treatment have been deposited under the accession numbers E-MTAB-13304 and E-MTAB-13328, respectively. Processed versions of these datasets can be found on Flow.bio: https://app.flow.bio/projects/710458278460049547. Targeted sequencing data from the reporter experiments have been deposited under the accession number E-MTAB-13329. Long-read sequencing data used to characterize the reporter system have been deposited under the accession number E-MTAB-13330. All iCLIP data from mESCs have been deposited under the accession number E-MTAB-13331. The MS proteomics data have been deposited to the ProteomeXchange Consortium via the PRIDE^78^ partner repository with the dataset identifier PXD066402.

## Code availability

All scripts for data analysis and figure generation (https://github.com/ulelab/interstasis-paper), and the GeRM package (https://github.com/ulelab/germ) are available on GitHub.
