# Supplementary material for: Collective homeostasis of condensation-prone proteins via their mRNAs
Source: Nature. 2025 Sep 24;647(8090):798–808. doi: 10.1038/s41586-025-09568-w (PMC12629991; doi:10.1038/s41586-025-09568-w)
Supplement: Supplementary file 1 — Supplementary Figs 1–3. [file 41586_2025_9568_MOESM1_ESM.pdf]

nature portfolio

https://doi.org/10.1038/s41586-025-09568-w

Supplementary information

Collective homeostasis of condensation-prone proteins via their mRNAs

In the format provided by the authors and unedited

Supplementary Information

Collective homeostasis of condensation-prone proteins via their mRNAs

Rupert Faraway[1,2,3,4,5#,*], Neve Costello Heaven[1,2,3*], Holly Digby[1,2,3], Klara Kuret Hodnik[6], Jure Rebselj[6], Oscar G. Wilkins[1,7], Anob M. Chakrabarti[1,8], Ira A. Iosub[1,2,3], Neža Vadnjal[6], Rhys Dore[2,3], Lea Knez[1], Stefan L. Ameres[4], Clemens Plaschka[5], Jernej Ule[1,2,3,6#]

1 The Francis Crick Institute, London, UK
2 UK Dementia Research Institute at King's College London, London, UK
3 Department of Basic and Clinical Neuroscience, Institute of Psychiatry Psychology & Neuroscience, King's College London, London, United Kingdom
4 Max Perutz Labs, University of Vienna, Vienna BioCenter, Vienna, Austria
5 Research Institute of Molecular Pathology, Vienna BioCenter, Vienna, Austria
6 National Institute of Chemistry, Ljubljana, Slovenia
7 Department of Neuromuscular Diseases, UCL Queen Square Institute of Neurology, UCL, London, UK
8 UCL Respiratory, Division of Medicine, University College London, London, UK

[#] Corresponding authors: Rupert Faraway <rupert.faraway@gmail.com>, Jernej Ule <jernej.ule@kcl.ac.uk>
* These authors contributed equally to this work

Supplementary Figure 1

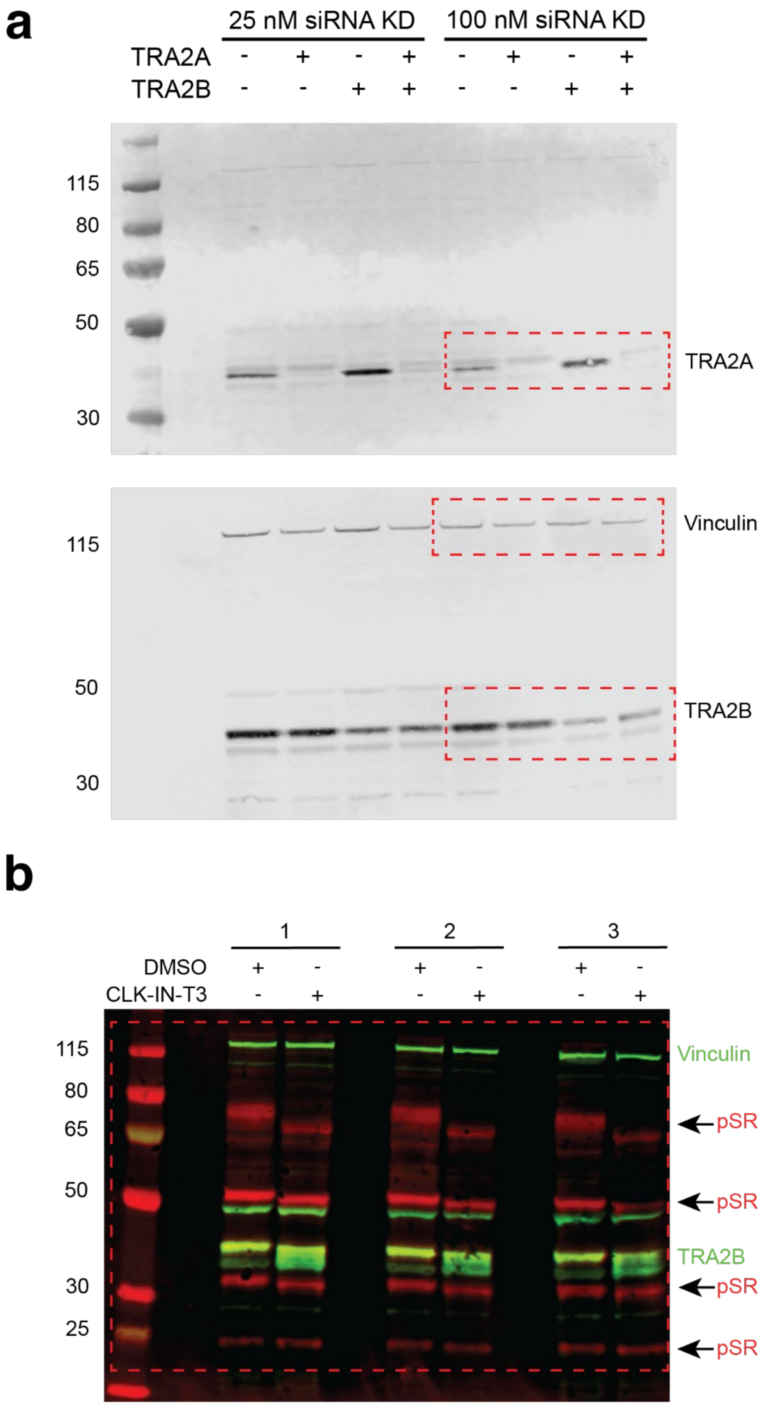

Supplementary Figure 1: Raw images of uncropped Western blot gels.

a, Uncropped gel for TRA2 siRNA knockdown Western blot. Upper gel shows TRA2A channel, lower gel shows TRA2B and vinculin loading control, which was run on the same gel. Regions shown in Extended Data Figure 8G are indicated by the red boxes. b, Uncropped gel for Western blot showing changes in TRA2B phosphorylation (green, lower band) and SR protein phosphorylation (red) following CLK-IN-T3 treatment. Vinculin loading control (green, upper band) was run on the same gel. The region shown in Extended Data Figure 10J is indicated by the red box.

Supplementary Figure 2

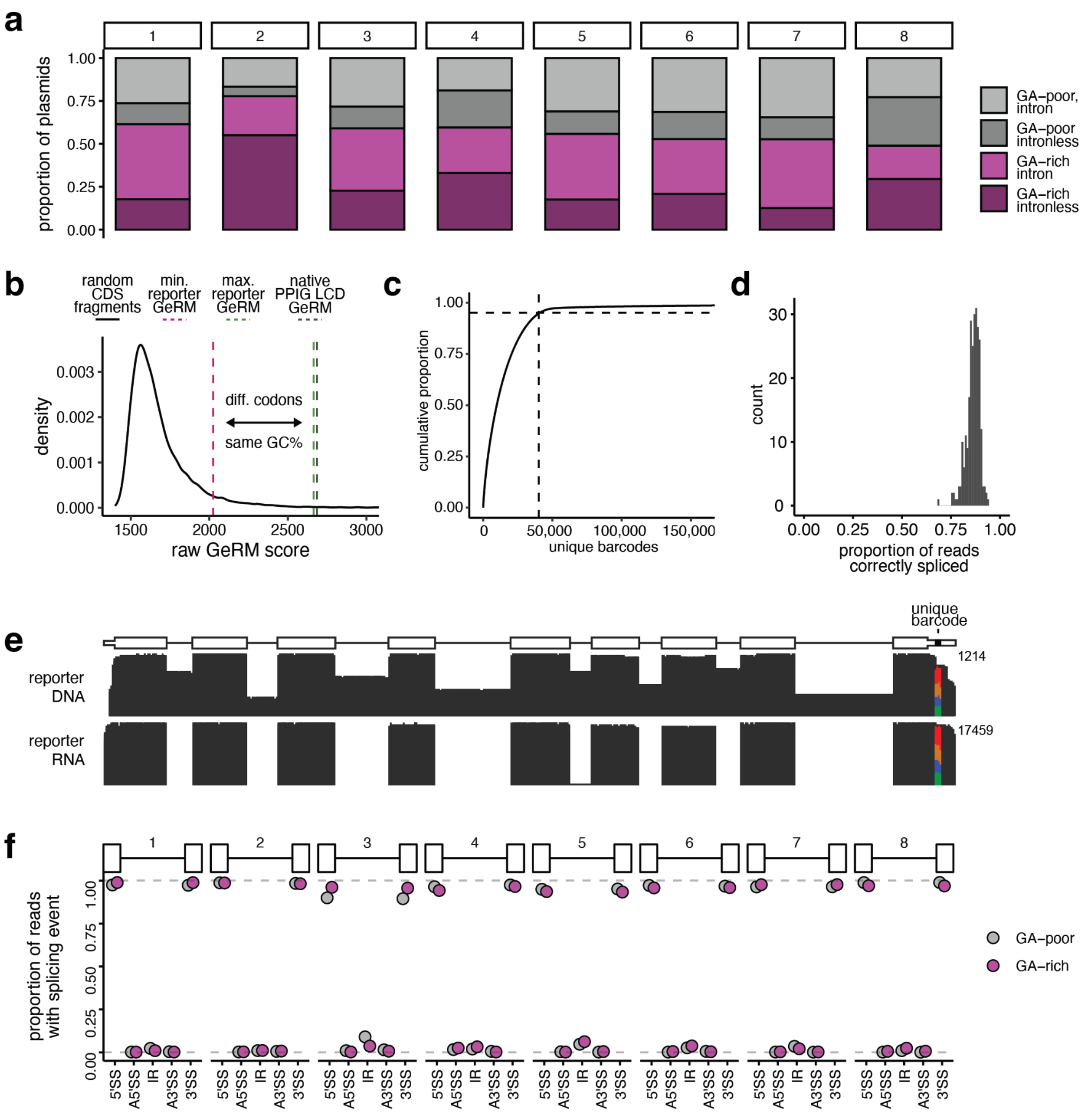

Supplementary Figure 2: Assembly and splicing of a combinatorial reporter construct.

a, The distribution of different sequence segment types in the reporter plasmid pool as determined by long-read sequencing. b, The distribution of total GeRM scores within randomly sampled CDS regions of the same size as $PPIG_{LCD}$. The black dashed line shows the GeRM total score of the native $PPIG_{LCD}$ sequence, while the pink and green dashed lines show the minimum and maximum total GeRM scores of the least and most multivalent $PPIG_{LCD}$ CDS from the reporter pool. c, The cumulative proportion of the total number of reporter barcode reads accounted for by unique reporter barcode sequences in a targeted sequencing experiment. The dashed lines represent the point at which 95% of barcode reads are accounted for. d, The proportion of long reads from reporter RNAs with correct splicing of all intended splicing junctions for all 256 different combinations of codon-biassed reporter genes. e, An example of the long read coverage for the reporter plasmids and the reporter RNA, showing efficient removal of introns. f, Quantification of splicing accuracy in long reads from reporter RNAs across all different possible intron junctions. 5'SS and 3'SS denote a correct splice site, while A5'SS and A3'SS denote incorrect junctions (upstream or downstream of the intended splice site). IR denotes intron retention.

Supplementary Figure 3

a

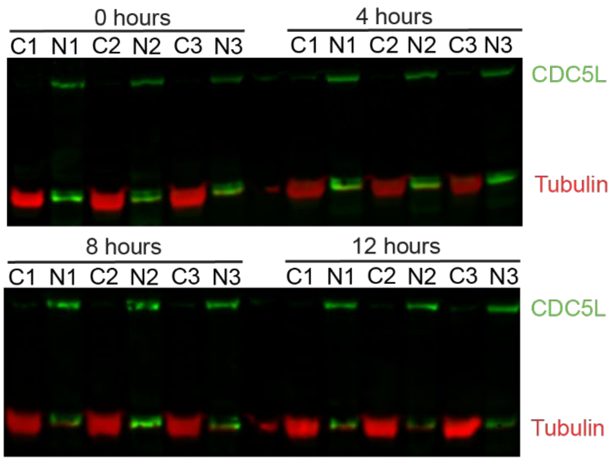

b

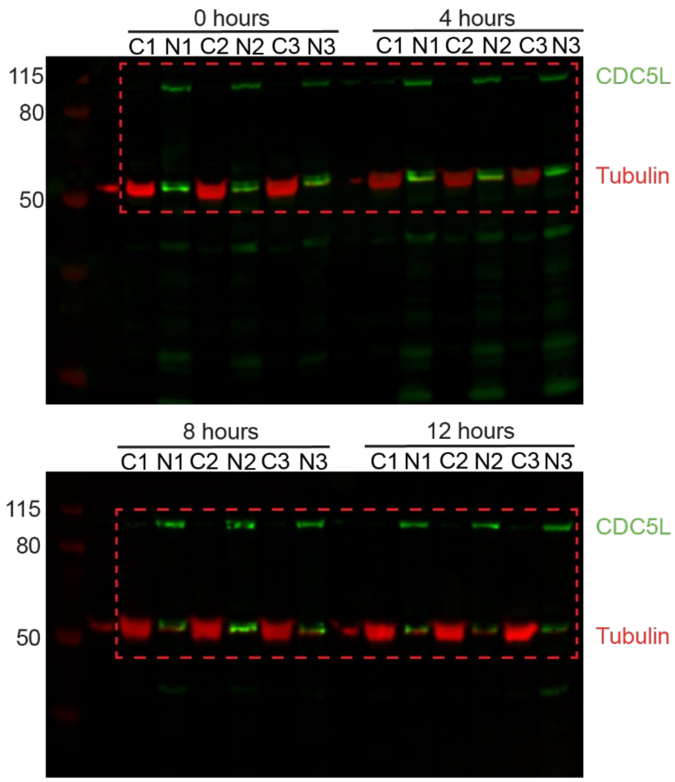

Supplementary Figure 3: Western blots exemplifying subcellular fractionation quality.

a, Western blotting of mScarlet-PPIG$_{LCD}$ cells incubated with doxycycline for 0, 4, 8 and 12 hour periods. Triplicates were prepared for each timepoint, with 'N' corresponding to the nuclear fraction and 'C' referring to the cytoplasmic fraction, each followed by a number indicating the replicate number. CDC5L (green) was used as a nuclear marker and alpha-tubulin (red) as a cytoplasmic marker. b, Uncropped gels with regions shown in part a indicated by the red boxes.
