# Supplementary material for: Collective homeostasis of condensation-prone proteins via their mRNAs
Source: Nature. 2025 Sep 24;647(8090):798–808. doi: 10.1038/s41586-025-09568-w (PMC12629991; doi:10.1038/s41586-025-09568-w)
Supplement: Supplementary file 2 — Reporting Summary [file 41586_2025_9568_MOESM2_ESM.pdf]

nature portfolio

Corresponding author(s): Jernej Ule, Rupert Faraway

Last updated by author(s): Jul 28, 2025

Reporting Summary

Nature Portfolio wishes to improve the reproducibility of the work that we publish. This form provides structure for consistency and transparency in reporting. For further information on Nature Portfolio policies, see our Editorial Policies and the Editorial Policy Checklist.

Statistics

For all statistical analyses, confirm that the following items are present in the figure legend, table legend, main text, or Methods section.

n/a	Confirmed	
☐	☒	The exact sample size (*n*) for each experimental group/condition, given as a discrete number and unit of measurement
☐	☒	A statement on whether measurements were taken from distinct samples or whether the same sample was measured repeatedly
☐	☒	The statistical test(s) used AND whether they are one- or two-sided<br>*Only common tests should be described solely by name; describe more complex techniques in the Methods section.*
☒	☐	A description of all covariates tested
☐	☒	A description of any assumptions or corrections, such as tests of normality and adjustment for multiple comparisons
☐	☒	A full description of the statistical parameters including central tendency (e.g. means) or other basic estimates (e.g. regression coefficient) AND variation (e.g. standard deviation) or associated estimates of uncertainty (e.g. confidence intervals)
☐	☒	For null hypothesis testing, the test statistic (e.g. *F*, *t*, *r*) with confidence intervals, effect sizes, degrees of freedom and *P* value noted<br>*Give P values as exact values whenever suitable.*
☒	☐	For Bayesian analysis, information on the choice of priors and Markov chain Monte Carlo settings
☒	☐	For hierarchical and complex designs, identification of the appropriate level for tests and full reporting of outcomes
☐	☒	Estimates of effect sizes (e.g. Cohen's *d*, Pearson's *r*), indicating how they were calculated

Our web collection on statistics for biologists contains articles on many of the points above.

Software and code

Policy information about availability of computer code

Data collection	ImageStudio 5 CLx (LI-COR); cellSens V4.2 (Olympus);
Data analysis	GeRM R package v0; nf-core RNA-seq NextFlow pipeline v3.12.0; rMATS v4.1.2; DBSCAN R package v1.1-11; tidyverse R package v2.0; HDMD R package v1.2; topGO R package v2.52; DESeq2 R package v3.17; Guppy v6.0.1; rapidfuzz python package v3.3.0; stringdist R package v0.9.10; apeglm R package v3.17, Cellpose v2.0; CellProfiler v4.2.6; FlowJO v9.9.6; R v4.2.0; STAR v2.7.0; UMItools v1.0; Ultraplex v1.2.5; Proteome Discoverer v3.1; BalancedRandomForestClassifier v0.12.37; cutadapt v4.4; UMAP v0.2.10.0<br><br>All scripts for data analysis available at https://github.com/ulelab/interstasis-paper<br>The GeRM R package is available, with installation and usage instructions, from the public GitHub repository: https://github.com/ulelab/germ

For manuscripts utilizing custom algorithms or software that are central to the research but not yet described in published literature, software must be made available to editors and reviewers. We strongly encourage code deposition in a community repository (e.g. GitHub). See the Nature Portfolio guidelines for submitting code & software for further information.

Data

Policy information about availability of data

All manuscripts must include a data availability statement. This statement should provide the following information, where applicable:

- Accession codes, unique identifiers, or web links for publicly available datasets
- A description of any restrictions on data availability
- For clinical datasets or third party data, please ensure that the statement adheres to our policy

All sequencing data has been deposited via ArrayExpress. 3' end sequencing experiments following PPIG-LCD expression and CLK-IN-T3 treatment are deposited under the accession numbers E-MTAB-13304 and E-MTAB-13328, respectively. Targeted sequencing data from reporter experiments are deposited under the accession number E-MTAB-13329. Long read sequencing data used to characterise the reporter system are deposited under the accession number E-MTAB-13330. All iCLIP data from mESC is deposited under the accession: E-MTAB-13331.The mass spectrometry proteomics data have been deposited to the ProteomeXchange Consortium via the PRIDE partner repository with the dataset identifier PXD066402.

Research involving human participants, their data, or biological material

Policy information about studies with human participants or human data. See also policy information about sex, gender (identity/presentation), and sexual orientation and race, ethnicity and racism.

Reporting on sex and gender	N/A
Reporting on race, ethnicity, or other socially relevant groupings	N/A
Population characteristics	N/A
Recruitment	N/A
Ethics oversight	N/A

Note that full information on the approval of the study protocol must also be provided in the manuscript.

Field-specific reporting

Please select the one below that is the best fit for your research. If you are not sure, read the appropriate sections before making your selection.

☒ Life sciences ☐ Behavioural & social sciences ☐ Ecological, evolutionary & environmental sciences

For a reference copy of the document with all sections, see nature.com/documents/nr-reporting-summary-flat.pdf

Life sciences study design

All studies must disclose on these points even when the disclosure is negative.

Sample size	No formal statistical methods were used to predetermine sample sizes. In most instances for quantitative experiments, we used at least 3 replicates per condition (with the exception of iCLIP, which was in duplicates). This level of replication is consistent with standard practice in the field, and has been sufficient in pilot studies and previous publications to detect reproducible and biologically meaningful differences with appropriate statistical power. For imaging-based quantification, an suitable number of fields of view (typically 8-10 per replicate) were analysed to ensure statistical robustness.
Data exclusions	No data was excluded.
Replication	All experiments were at least duplicated, if not replicated more times, in independent experiments.<br>All attempts at replication were successful.
Randomization	No randomisation was performed, due to the nature of the experiments. Treated samples and controls were prepared under the same conditions and compared to each other.
Blinding	Blinding was not performed in this study due to the nature of the experimental design. This involved clearly distinguishable conditions, particularly in the case of imaging data collection, which made blinding impractical. To minimise potential bias, all image acquisition parameters were standardised across conditions, and quantitative analyses were performed using automated pipelines, with consistent thresholding criteria applied across all samples.

Reporting for specific materials, systems and methods

We require information from authors about some types of materials, experimental systems and methods used in many studies. Here, indicate whether each material, system or method listed is relevant to your study. If you are not sure if a list item applies to your research, read the appropriate section before selecting a response.

Materials & experimental systems

n/a	Involved in the study
☐	☒ Antibodies
☐	☒ Eukaryotic cell lines
☒	☐ Palaeontology and archaeology
☒	☐ Animals and other organisms
☒	☐ Clinical data
☒	☐ Dual use research of concern
☒	☐ Plants

Methods

n/a	Involved in the study
☒	☐ ChIP-seq
☒	☐ Flow cytometry
☒	☐ MRI-based neuroimaging

Antibodies

Antibodies used	Primary antibodies: rabbit anti-TRA2B (Abcam, ab31353, 1:1000) rabbit anti-SON (Sigma, HPA023535, 1:1000) mouse anti-SC35 (Sigma, S4045, 1:500) mouse anti-TRA2A (Novus Biologicals, H00029896-B01P, 1:500) rabbit anti-vinculin (ThermoFisher, 700062, 1:2000) mouse anti-phosphoepitope SR proteins (Sigma, MABE50, 1:1000)<br>Secondary antibodies: Goat pAb anti-Rabbit IgG Alexa Fluor 647 (Abcam, ab150079, 1:500) Goat pAb anti-Rabbit IgG Alexa Fluor 488 (Abcam, ab150077, 1:500) Goat pAb anti-Mouse IgG Alexa Fluor 488 (Abcam, ab150113, 1:500) Goat pAb anti-Mouse IgG Alexa Fluor 647 (Abcam, ab150115, 1:500).
Validation	All antibodies are validated by the manufacturer, and validation information and quality certificates can be obtained from the manufacturer's websites. anti-SON is a Sigma Prestige Antibody which has undergone independent enhanced validation (see https://www.sigmaaldrich.com/GB/en/technical-documents/technical-article/protein-biology/immunohistochemistry/antibody-enhanced-validation) for ICC/IHC in humans by the Human Protein Atlas (HPA) project. anti-SC35 has been validated by Sigma in ICC, IP and EM across multiple species including human. anti-TRA2B has been validated for ICC, WB and IP in human and mouse according to manufacturer website and across numerous peer-reviewed publications. anti-TRA2A is validated by the manufacturer via knockdown for use in ICC/IF and Western blot in human and mouse samples. anti-vinculin and anti-phosphoepitope SR proteins have been validated by ThermoFisher and Signma respectively for Western blotting.

Eukaryotic cell lines

Policy information about cell lines and Sex and Gender in Research

Cell line source(s)	Cells lines (HeLa, mESC) were obtained from the Francis Crick Institute's Cell Science Science Technology Platform (STP). HeLa cells are of human origin, and were derived from a patient of female sex.
Authentication	Cell line authentication performed by Francis Crick Institute's Cell Science Science Technology Platform (STP) using PCR with a multiplex of species specific primers. HeLa cells with verified on Human STR (S58/20 & S64/20) using Promega 16HS to PCR and fragment analysis on the 3500XL Applied Biosystems Genetic analyser. Profile obtained was then matched with that for HeLa on the online Cellosaurus database.
Mycoplasma contamination	Cell lines tested negative for mycoplasma contamination.
Commonly misidentified lines<br>(See ICLAC register)	No commonly misidentified cell lines were used in this study.

Plants

Seed stocks	N/A
Novel plant genotypes	N/A
Authentication	N/A
