# Supplementary material for: Collective homeostasis of condensation-prone proteins via their mRNAs
Source: Nature. 2025 Sep 24;647(8090):798–808. doi: 10.1038/s41586-025-09568-w (PMC12629991; doi:10.1038/s41586-025-09568-w)
Supplement: Supplementary file 3 — Supplementary Tables 1–9 and a Supplementary Table guide. [file 41586_2025_9568_MOESM3_ESM.zip › SI_Guide.pdf]

Supplementary Information Guide

Collective homeostasis of condensation-prone proteins via their mRNAs

Rupert Faraway[1,2,3,4,5#,*], Neve Costello Heaven[1,2,3*], Holly Digby[1,2,3], Klara Kuret Hodnik[5], Jure Rebselj[5], Oscar G. Wilkins[1,6], Anob M. Chakrabarti[1,7], Ira A. Iosub[1,2,3], Neža Vadnjal[5], Rhys Dore[2,3], Lea Knez[1], Stefan L. Ameres[4], Clemens Plaschka[5], Jernej Ule[1,2,3,6#]

1 The Francis Crick Institute, London, UK
2 UK Dementia Research Institute at King's College London, London, UK
3 Department of Basic and Clinical Neuroscience, Institute of Psychiatry Psychology & Neuroscience, King’s College London, London, United Kingdom
4 Max Perutz Labs, University of Vienna, Vienna BioCenter, Vienna, Austria
5 Research Institute of Molecular Pathology, Vienna BioCenter, Vienna, Austria
6 National Institute of Chemistry, Ljubljana, Slovenia
7 Department of Neuromuscular Diseases, UCL Queen Square Institute of Neurology, UCL, London, UK
8 UCL Respiratory, Division of Medicine, University College London, London, UK

[#] Corresponding authors: Rupert Faraway <rupert.faraway@gmail.com>, Jernej Ule <jernej.ule@kcl.ac.uk>
* These authors contributed equally to this work

Table of Contents

Supplementary Figure 1...1
Raw images of uncropped Western blot gels.

Supplementary Figure 2...3
Assembly and splicing of a combinatorial reporter construct.

Supplementary Tables (as separate files)

Supplementary Table 1
Species used for analysis of R codons usage in R-LCDs.

Supplementary Table 2

Details of multivalency reporter pool construction and primer sequences used for targeted sequencing library preparation.

Supplementary Table 3

Quantification of barcodes and gene structures from all targeted sequencing experiments using the multivalency reporter.

Supplementary Table 4

DESeq2 analysis output from all 3' end sequencing experiments, with additional gene information used for classification.

Supplementary Table 5

Sequences of LUC7L3 reporter plasmids.

Supplementary Table 6

All GeRM CDS regions used for analysis of multivalency.

Supplementary Table 7

All LCD regions used for analysis of multivalency and human codon bias.

Supplementary Table 8

Features used for prediction of retained mRNAs upon PPIG-LCD expression.

Supplementary Table 9

Binding potential scores for all RBPs and all reporter sequences.
